# Defining Environmental Health Literacy

**DOI:** 10.3390/ijerph182111626

**Published:** 2021-11-05

**Authors:** Marti Lindsey, Shaw-Ree Chen, Richmond Ben, Melissa Manoogian, Jordan Spradlin

**Affiliations:** 1 Southwest Environmental Health Sciences Center, College of Pharmacy, University of Arizona, Tucson, AZ 85721, USA; richmond@pharmacy.arizona.edu (R.B.); melissakmanoogian@gmail.com (M.M.); Spradlij@oregonstate.edu (J.S.); 2 Quality & Safety Institute, Rochester Regional Health, Rochester, NY 14617, USA; shaw-ree.chen@rochesterregional.org

**Keywords:** environmental health, literacy, knowledge, skills, risk communication, disease prevention

## Abstract

“Environmental Health Literacy” (EHL) is embraced as important for improving public health by preventing disability and disease from our environment. This study aimed to determine knowledge and skill items identified by Environmental Health (EH) professionals as being associated with EHL and to understand how these items rank by importance. Such a coordinated effort to tease out skills and knowledge needed for EHL had not previously been made. We utilized a mixed-methods approach of semi-structured interviews of 24 EH professionals and a quantitative survey with 275 EH professionals across the United States. Interviews identified 37 skill and 69 knowledge items, which were used to create the survey questions. Survey results indicate 32 knowledge items and six skill items considered essential by >50% of respondents where consensus was reached between professional groups (chi square test: *p* > 0.05). We further identified six knowledge items, which >70% of EH professionals agreed were essential for EHL. The identification of these knowledge and skill items sets the stage for further research that includes exploring agreement with more diverse stakeholders, developing comprehensive measures of EHL and evaluation of methods and materials designed to improve EHL.

## 1. Introduction

The NIEHS Environmental Health Sciences (EHS) Core Centers Program [1] funds institutional infrastructure to support scientific equipment, facilities, and other resources that can be shared among environmental health researchers. By pursuing shared research questions, the EHS Core Centers identify emerging issues that advance understanding about how pollutants and other environmental factors affect human biology and may lead to disease. The Environmental Health Sciences Core Center Program includes Community Engagement Cores (CECs) [2] to foster community–university partnerships. Each CEC builds and sustains a dialog between the center and its defined audience, helping researchers understand the environmental health questions of the community and community members understand the environmental health research of each center. The NIEHS offers supplemental funds to Core Center grants to promote collaboration to investigate issues of importance to research and community engagement for the NIEHS community. This study was conducted in a collaboration between the Community Engagement Cores (CECs) of the University of Arizona, Southwest Environmental Health Sciences Center [3], and the University of Rochester Environmental Health Sciences Center [4], with supplemental funding from the NIEHS.

### 1.1. Framework for Researching Environmental Health Literacy 

The process and methodology by which CECs communicate about environmental health research is informed by our understanding of Environmental Health Literacy (EHL). In 2014, the National Institute of Environmental Health Sciences (NIEHS) described environmental health literacy (EHL) as “an emerging and evolving concept that bridges shared theories from the fields of risk communication, environmental health science, behavioral science, evaluation, communications, public health, and the social sciences. The process of becoming environmentally health literate entails raising scientific literacy, environmental literacy, and numeracy among the general public while increasing awareness of specific exposures and their potential health effects” [5]. CECs and other organizations working with community groups would benefit greatly from a shared understanding of what skills and knowledge underpin EHL. The research reported here describes the outcomes of an effort to describe the knowledge and skills of being environmentally health literate, building on health literacy, science literacy, and environmental literacy. 

### 1.2. Health Literacy 

Although health literacy (HL) was not included in the 2014 EHL definition, we feel that HL contributes to EHL. A systematic review described HL as having three domains: health care, disease prevention, and health promotion [6]. Within the domain of health promotion, a health literate person should be able to inform themselves about detriments to health in their social and physical environments, understand the information obtained, interpret and evaluate the information, and make informed decisions about the health detriments [6]. The concept of HL has been well studied and is defined as the ability to obtain and comprehend healthcare information, and use that information to obtain health services, make appropriate health decisions, and follow instructions for treatment [7,8]. The relationship between weak literacy skills and poor health outcomes is now well supported by data from many developed nations [9,10]. 

As more professionals place greater attention on the HL of those within various communities they serve, strategies to promote literacy, numeracy, and language skills in populations are developing to improve health communications [9]. Although the traditional focus of HL was previously placed on the management of words and numbers in a medical setting, it has, in recent years, expanded to also include more complex skills such as information seeking, decision making, problem solving, critical thinking, and effective communication within public health [11]. It has been determined that these skills are essential for an individual to obtain a deeper understanding of their health and the health of their population. 

Many studies have shown a link between health inequities and the HL of populations [9,10,11,12,13,14,15,16,17,18]. Although HL tends to focus on an individual’s ability to use knowledge to improve or protect their health, it is important to acknowledge the importance of community and population HL [18]. An individual with greater HL can only do so much. However, if there is a deeper understanding of health and a greater rate of HL at the community or population level, it is likely that the existing health inequities will decrease. One study found that promoting and providing the prerequisites of health (education, income, social justice, and equity), increasing the health coverage, and empowering patients in the health care setting led to a widespread increase in HL among the population as a whole [17]. Such promising results outline possible future interventions to achieve a greater HL across multiple populations. 

Insofar as environmental exposures represent detriments to health in the physical environment, HL should overlap with EHL. However, HL does not address how knowledge is translated from laboratory, public health, or field research to actions that improve the health of populations exposed to environmental hazards and contaminants. This is better described as Science Literacy, which is expanded upon below.

### 1.3. Science Literacy

The knowledge of how a scientific framework is used by people to make decisions, based on fact, research, and knowledge is the foundation of Science Literacy (SL). Science and technology are essential components of everyday life for most individuals and offer a platform on which to promote economic growth and welfare for individuals in society [16,17,18,19]. To become responsible, contributory, and healthy citizens of society, individuals must be able to contribute to the daily conversations and societal debates surrounding environmental health and science. The future of the environment, population health, and sustainability all depend on this ability of individuals to partake in science discussion with a strong and supportive scientific literacy [20]. Strategies to build SL include focusing on reading, writing, and communication skills, but many schools fall short of optimal science-based reading instruction [21,22]. Although there are more aspects included in science literacy than just reading, it is important for teachers to incorporate science reading into lesson plans to better equip students for future science discussions and reading in everyday life. Although the joining of SL and HL appears to serve as a pre-existing description of EHL, and provides environmental professionals with a framework to train teachers and provide opportunities for students to explore how the environment impacts human health [23], there is a critical aspect of environmental health that is not incorporated in SL and HL alone, namely, environmental justice. 

### 1.4. Environmental Justice

Environmental justice (EJ) is rooted within the civil rights movement, with the EJ movement specifically emerging from Warren County, North Carolina, where the local communities struggled with toxic contamination in the 1970s–1980s [24]. Although the Warren County experience is accredited as the birth of the EJ movement, low-income and minority communities have long been, and continue to be, impacted by toxic contamination. Another well-known example concerns trichlorethylene (TCE) which, in the 1950s, was commonly dumped in open pits, drains, and directly on the ground, contaminating South Tucson, a community largely consisting of Latinx and Native American families [24]. 

Environmental injustice is classified as unequal access to healthy and clean environments, including environmental amenities [18]. One national study of urban areas across the U.S. found that nearly one-third of low-income urban communities contained hazardous waste facilities [21]. The current literature surrounding EJ consistently shows that environmental risks, present or potential, tend to be distributed inequitably, largely impacting racial and ethnic minority communities and communities with lower socioeconomic status [17]. Unequal exposure to environmental hazards for low-income and minority communities continues to be a major challenge across the country and indeed internationally [25,26,27,28,29,30,31]. 

Studies continue to emerge, thus solidifying a link between minority status, low socioeconomic status, and community proximity to toxic landfills [18]. In neighborhoods containing commercial waste facilities, 56% of the population are people of color, and the poverty rate within such communities continues to be 50% higher than in communities without commercial waste facilities [21]. Therefore, community engagement, outreach, and education are essential for such communities.

### 1.5. Environmental Health Literacy

Environmental Health Literacy (EHL) has been an emerging field since the 1950s, and NIEHS had an influential role in producing programs to expand the EHL of populations in the early 1990s [3,22]. EHL is an area of study that combines elements from different disciplines, including health literacy, risk communication, environmental health, communications research, and safety culture [22]. The basics of EHL start when an individual understands the link between environmental exposures and health outcomes. However, the entirety of EHL includes many complex topics, including those described above.

A gap has been acknowledged regarding environmental knowledge, environmental awareness, and the ability to display pro-environmental behavior [31]. No definitive explanation has been discovered to explain this gap between knowledge and action, yet many factors are taken into consideration for causing this gap including, but not limited to, demographic factors, external factors (e.g., economic, social, cultural), and internal factors (e.g., motivation, awareness, priorities) [31]. 

EHL is a field with great potential that is still being discovered as more professionals enter the environmental health field to improve the health of communities and populations around the world. The field of Community Engagement Cores of the NIEHS [1,22] views elevating the EHL of populations as providing individuals a chance to take control of their own health, and be aware of how their actions may affect the environment around them. As the science develops further, it is critical to recognize the importance of EHL’s impact on the health of the populations and the environment. 

The use of EHL education to effectively communicate environmental risks has shown a positive impact in protecting communities disproportionately exposed to environmental hazards [19]. With enhanced EHL, individuals and communities might make better informed decisions about environmental risks to benefit their overall health. Enriched and expanded EHL education is important to share with individuals, specifically from communities of color and poor communities. Gaining understanding of how the environment impacts health creates knowledge, power, and the agency necessary to enact positive change and social action to combat environmental injustices [23]. 

This study aimed to identify knowledge and skill items identified by Environmental Health professionals as being associated with EHL, and understand how these items rank by importance. The authors propose that an increased understanding of these knowledge and skill items may lead to standardized interventions and measurements in the field of environmental health literacy, which would then lead to greater ability of people and populations to understand and advocate for mitigation of environmental hazards. 

### 1.6. Defining Environmental Health Literacy Project 

The aim of this project was to produce a consensus definition of EHL, using the perspectives of four professional groups that have an interest in environmental health information: clinicians, environmental health (EH) researchers, environmental public health educators, and users of EH information in the public. Gathering input from community members, students, or leaders was viewed as beyond the scope of the funding available. The aims of this project were explored using a mixed-methods approach.

## 2. Materials and Methods

The first objective was to identify a broad family of knowledge and skills through in-depth interviews with researchers, clinicians, environmental health educators, and community partners. The second objective was to focus on this broad family of competencies using a survey process to identify those competencies that had most agreement from participants as being essential to EHL. 

Interviewees were asked open-ended questions including: (1) How participants personally and professionally manage environmental exposures, (2) How participants define an “effective response” to chronic and acute exposures, and (3) What participants think are characteristics that define an individual who is capable of effective understanding and response to environmental exposures. 

The themes chosen for the survey questions were EHL competencies that were deemed applicable to a national audience. The survey was distributed to NIEHS [1] grantees nationwide. Invitations to participate in the survey were distributed via email to Core Center community engagement staff, partners of the research team, and Core Center directors, researchers, and clinicians. Survey questions fell into 5 categories: (1) identifying exposures and resultant health outcomes, (2) risk management, (3) judging reliability of information, (4) numeracy, and (5) communication. 

### 2.1. Interviews

#### 2.1.1. Interview Process and Content

Interviews were conducted with 24 EH professionals, half by the University of Arizona and half by University of Rochester team members. The groups targeted by each collaborator were chosen based on existing partnerships and ongoing projects at each site. A semi-structured guide was developed to focus the interview process on how EH professionals defined environmental health and what skills and knowledge were important for understanding environmental health issues. The questions can be viewed in Supplementary A: Defining Environmental Health Literacy—Interview Script. Interviews took, on average, 60 min. Participants provided verbal consent and were given a USD 20 incentive VISA card. All interviews were audio-recorded and transcribed verbatim. 

Purposive sampling was employed to ensure participants represented a broad range of environmental health professionals. We sought the following types of professionals: (a) individuals who are engaged in EH outreach and education; (b) secondary school educators who have taught at least one semester/quarter of a course where EH was a topic; (c) EH basic researchers and epidemiologists; and (d) physicians who have counseled patients on EH concerns. Most interviewees (n = 24) were female, white, with a Master’s degree and/or a Ph.D. They were researchers or educators. The interviewees had a mean age of 50 years. 

#### 2.1.2. Analysis of Interview Transcripts

Interviews were recorded and transcribed verbatim. The transcripts were analyzed using a grounded theory approach. After each interview, the transcripts were reviewed by the person who conducted the interview, and evaluated for knowledge items and skill items. Quotations were selected to represent comments from interviewees as fitting into the themes. Transcripts were then exchanged with another person on the team, to retag the interviews and allow for agreements. Where there was not agreement, the section of the transcript was further reviewed by the entire team. Comments were labeled if they were not relevant to EHL skills and knowledge. After exchanging transcripts, the entire team met to make suggestions for existing or new tags, or to confirm agreement with existing tags/themes. New knowledge/skills were added to an overall list of themes and tags. Emergent themes were discussed in depth among the team to develop a coding framework and to guide avenues of exploration in subsequent interviews. Interviews continued until data saturation was reached, i.e., when no new themes emerged, in twenty-four interviews.

In this approach, key points that arose during the interviews were marked with a series of codes, which were extracted from the text. The codes were grouped to form categories [32]. Two sets of analyses were conducted, one at the institution where the interviews were conducted, and one at the collaborating institution. This process of “researcher triangulation,” in which more than one researcher analyzes the same data, produces rigorous data as their different perspectives serve to confirm the developing themes [33,34]. A total of 106 common themes relating to skills and knowledge required for identifying, understanding, and taking action regarding environmental exposures were identified from these interviews. 

### 2.2. Survey

#### 2.2.1. Survey Process and Content

The survey items were a refinement of the common themes that emerged from Objective 1. The goal was to obtain responses from 75% of centers participating with an anticipated total of over 50 responses, which was exceeded with 215 participants completing the survey. The 106 themes, tags, and quotations were used to develop 105 knowledge and skill items for a subsequent quantitative survey. A sociological linguist was consulted to ensure that items correctly reflected knowledge and skills as described in the interviews. The survey was created in REDCap and hosted at the University of Rochester. Eight EH community engagement professionals [5] were consulted to pilot the survey format and usability. The survey took between 30 and 40 min to complete.

A web-link to the final survey was sent to the Partners for Environmental Public Health (PEPH) Network [35] listserv, which reaches outreach professionals and researchers who are officially associated with the National Institutes of Environmental Health Sciences (NIEHS) [36] via grants. PEPH is a network of scientists, community members, educators, healthcare providers, public health officials, and policymakers who share the goal of increasing the impact of environmental public health research at the local, regional, and national level.

Of the 215 participants who completed the survey at least partially, 40 completed less than 80% of the items. These 40 surveys were removed from further analysis. The majority of survey respondents were female, white, with a Master’s degree and/or a PhD, and held research positions. The survey respondents had a mean age of 47.5 years. The 175 respondents were asked to report which of 6 professions they most identified with: Basic Science Researchers, Other Type of Researchers, Educators, EH Outreach Specialists, Medical Doctors, or Other Professions. The first 150 survey participants received a USD 20 electronic gift card. 

The survey asked respondents to think of the “environmental health literate individual” as someone who is “functionally literate”, that is, the level at which people have enough reading ability to function in their lives, at minimum, and able to read and comprehend information that is conveyed on the reading level of a 4–6th grader (comparable to the front page of a newspaper), has knowledge of how the environment and health are connected, and can respond rationally and logically to environmental health information delivered through books and pamphlets, the Internet, the news, or by other people. They were asked to judge each item in the survey independently using the following scale:

- ESSENTIAL: You believe these knowledge and skills are essential for the lay person to make decisions about themselves and their family. These are the core knowledge and skills that all EHL people should have;
- INTERMEDIATE: You believe these knowledge and skills go beyond what is essential for a lay person. They might make them more effective at communicating or rationally advocating their stance;
- EXPERT: You believe these knowledge and skills are important for the lay person to be able to deal with environmental health experts, or to contribute to decision making/policy processes within their community;
- NOT INCLUDED: You believe these knowledge and skills are not a part of EHL.

#### 2.2.2. Analysis of Survey Data

The survey was conducted using REDCap, which generated data that was analyzed by the Data Science Facility Core of the SWEHSC. The surveyed asked whether 105 EH knowledge and skill items are essential to basic EH literacy or only required by experts, intermediates, or not at all. The data had 281 rows and 338 columns. These represented 281 responses to the Environmental Health (EH) Literacy Survey. There were 66 completely blank responses, which were believed to be caused by surveys being opened, but never started. These were removed from the analysis. There were 40 cases in which there were half or fewer responses to fewer than 80% of the items. The team decided to also remove these cases. The analysis was conducted on 175 survey respondents.

A χ^2^ test was used to assess the agreement among all 175 professions for the highest ranked items. This was accomplished by creating an Essentialness variable by consolidating the Intermediate, Expert, and Not Included responses into a Non-Essential category. A 5 × 2 contingency table was created for each item, where the rows are the professions, and the columns are Essential or Non-Essential. The χ^2^-test assessed the independence between these two variables (e.g., profession and essentialness), where the null hypothesis was that the variables are independent.

The analysis was summarized in net-stacked bar plots. They show, for each item, the percent of responses to each of four choices (Essential, Expert, Intermediate, and Not Included). The plots consider Essential as a positive response and the others as negative. In other words, Essential is required for basic EH literacy, and the other 3 choices give some indication that it is not. The numbers next to the bars indicate the item number. The items were simply numbered from 1 to 105. Note that items 1 to 69 are knowledge-type items and items 70 to 105 are skill-type items. Plots are broken down by knowledge and skill items, and later by the 13 categories of questions found in the survey (e.g., General Environmental Health Knowledge, Knowledge about exposures and environmental agents). The entire Data Science Report is in Supplementary B: Environmental Health Literacy Data Analysis.

## 3. Results

### 3.1. Results of Interviews

A total of 69 knowledge items and 37 skill items were identified through the interviews with EH professionals. They were clustered into 13 categories of questions.

1. General EH Knowledge
2. Exposure and Environmental Agents
3. Knowledge of Risks and Hazards
4. General Knowledge about Biology
5. Knowledge about the Media
6. Knowledge about EH Research
7. Knowledge about Policy
8. Knowledge about Sources of Information
9. Knowledge about Exposures and Resultant Outcomes
10. Risk Management
11. Judging Reliability
12. Numeracy
13. Communication.

### 3.2. Results of the survey

This study identified 32 knowledge and six skill items agreed upon by EH professionals as being essential for EHL by at least 50% of the respondents. The lists of essential items are displayed below in tables and bar plot graphs. The report from the statistician can be viewed in Supplementary B: Environmental Health Literacy Data Analysis. The data derived from the survey is shown in two ways: in tables that identify the Essential survey items and in graphs that indicate the number of respondents who chose the item to be Essential to EHL (coral), Expert EHL (green), or Intermediate EHL (turquoise). “Essential” was regarding as a positive response, whereas all other choices were negative. The consensus group was comprised of the 175 experts who completed the entire survey. 

### 3.3. Tables and Figures

In Table 1: Highest Cluster of Essential Knowledge Items and Table 2: Highest Cluster of Essential Skill Items, each item is listed along with the percent of respondents that identified the item as being essential, and the *p*-value for the chi-square test, signifying the level of agreement between different occupational groups. A *p*-value of greater than 0.05 indicates significant agreement between professional groups; thus, the item was considered to have reached consensus.

**Figure 1:**
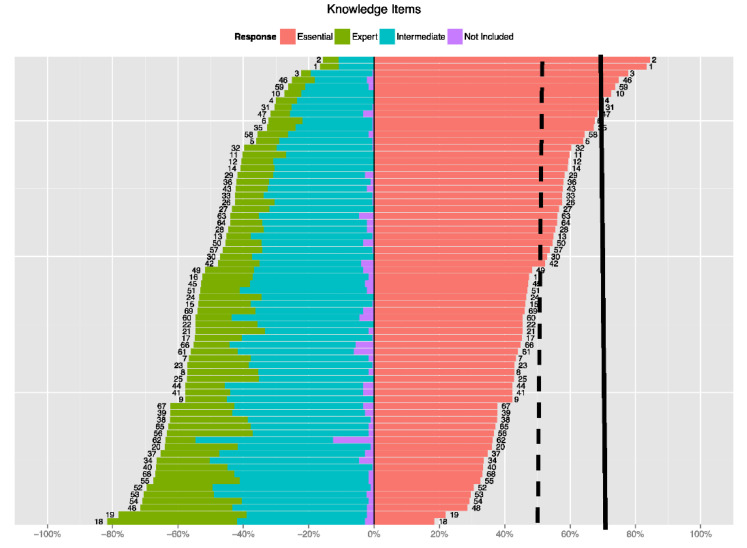
Distribution of the Knowledge Items.

**Figure 2:**
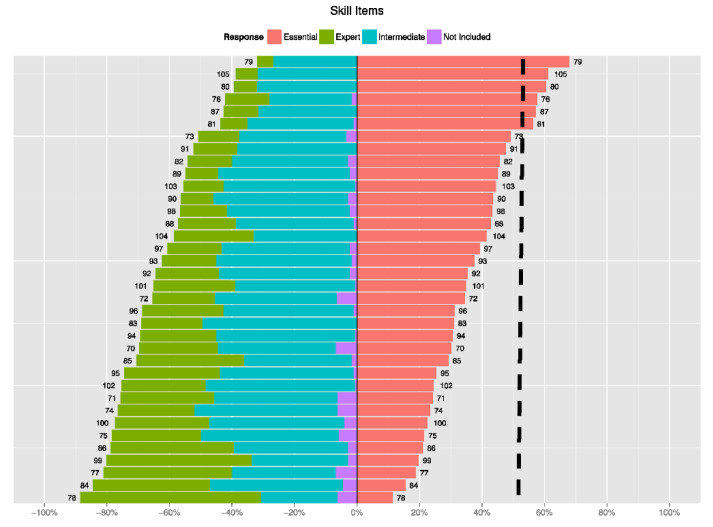
Distribution of the Skill Items.

Figure 1: Distribution of the Knowledge Items and Figure 2: Distribution of Skill *Items* contains the bar plot graphs of items ranked by survey participants, with vertical lines showing the number who agreed the item was Essential. Plots were used to visually identify clusters of items that were highly ranked among all respondents. The vertical lines indicate the percentage of respondents who agreed about the designation of Essential.

#### 3.3.1. Results: Knowledge Items

Table 1 shows the 32 knowledge items that were deemed to be Essential to EHL by >50% of the respondents to the survey. They are arranged by the percentage of the respondents who ranked them as Essential and the p-Value indicating the independence between profession of the respondent and essentialness.

**Table 1:** Highest Cluster of Essential Knowledge Items.

Knowledge Item.	% Ranked Essential	χ^2^ *p*-Value
Knows that “environment” includes everything around us: air, water, man-made chemicals, natural chemicals, other people, culture, animals, food, microorganisms and more.	84.40%	0.662
Knows that people affect the health of the environment.	83.40%	0.974
Knows that the environment affects every individual person’s health.	77.70%	0.894
Knows that media coverage can be unbalanced.	74.90%	0.967
Knows that an individual citizen can influence government policy.	73.70%	0.663
Knows that the environment can affect a person’s health through exposure to many different environmental agents.	72.60%	0.745
Knows that the environment affects the health of the population.	69.90%	0.343
Knows that complete avoidance of environmental agents is probably not possible; however, a person may be able to control their own exposure through individual behaviors.	69.50%	0.585
Knows that media may use sensationalism in reporting environmental hazards or risks.	68.40%	0.824
Knows that the environment affects people’s health in long-term ways.	67.60%	0.279
Knows that the term “hazard” refers to an environmental agent that can potentially harm a person’s health.	67.20%	0.885
Knows that the presence of environmental agents in commercial products, buildings, water and air is regulated by government policy.	64.40%	0.947
Knows that the environment affects people’s health in short-term ways.	64.00%	0.498
Knows that a small amount of an environmental agent may have a very big effect, depending on the nature of the agent.	60.30%	0.303
Knows that the environment can affect a person’s health through cumulative exposures to a particular environmental agent.	59.80%	0.359
Knows that environmental agents enter a person’s body and affect their health through 3 main ways: eating, breathing, and absorption through skin.	59.40%	0.66
Knows that a person’s exposure to environmental agents can be chronic and subtle, getting a little exposure over a long period of time.	59.20%	0.606
Knows that moderation in terms of eating foods or using commercial products is, in general, a good practice to minimize the negative effects of environmental agents.	58.30%	0.928
Knows that the term “risk” refers to the chance or probability that a person will be harmed or will experience an adverse health effect if exposed to a hazard.	57.90%	0.224
Knows that a news headline may not reflect an article’s actual content and should not be taken out of context.	57.70%	0.362
Knows that the term “dose” refers to how much of something goes into a person’s body	57.50%	0.579
Knows that the term “frequency of exposure” refers to how often a person’s body gets exposed to an environmental agent.	56.60%	0.111
Knows that sources produced by for-profit businesses and organizations may reflect interests and agendas and be less objective and reliable.	56.10%	0.421
Knows that reliable sources of information tend to provide supporting evidence.	56.00%	0.747
Knows that one way of minimizing dose and frequency of exposure to environmental agents in foods and commercial products is to practice moderation and diversity in eating.	55.40%	0.726
Knows that a person’s exposure to environmental agents can be acute, getting a lot of exposure in a short period of time.	54.90%	0.506
Knows that research can take a long time, and that’s normal.	54.60%	0.306
Knows that environmental agents that cause harm to the health of an animal are likely to cause harm to human health as well.	53.70%	0.534
Knows that the ability of an environmental agent to cause negative health effects depends on a person’s health and their age and stage of development.	52.90%	0.121

Higher *p*-values indicate more agreement.

Figure 1: Distribution of the Knowledge Items contains a graph of the knowledge items ranked by survey participants. It indicates the number of respondents who chose the item to be Essential to EHL (coral), Expert EHL (green), or Intermediate EHL (turquoise). Dashed vertical lines indicate >50% of respondents agreed about the designation of Essential. The solid line indicates >70% of respondents agreed the top six items are essential, which was considered strong agreement. The vertical dashed line shows agreement of >50% of the respondents and the vertical solid line shows agreement of >70% of the respondents.

#### 3.3.2. Results: Skill Items

Table 2 Highest Cluster of Essential Skill Items shows the skill items that were deemed to be essential by >50% of the respondents to the survey. 

**Table 2:** Highest cluster of essential skill items.

Skill Item	% Ranked Essential	χ^2^ *p*-Value
Is able to find info explaining how to reduce risks in their life	68.00%	0.625
Is able to convey their concerns about environmental risk to others	61.20%	0.595
Is able to find info about hazards in their microenvironment, such as at home or in the workplace	60.60%	0.661
Is able to find info about regional/community environmental hazards/issues	60.60%	0.661
Is able to identify well-known/ established hazards in their environment	57.70%	0.194
Is able to judge whether an information source is reliable	57.30%	0.164

Higher *p*-values indicate more agreement.

Figure 2: Distribution of the Skill Items contains a graph of the skill items ranked by survey participants. It indicates the number of respondents who chose the item to be Essential to EHL (coral), Expert EHL (green), or Intermediate EHL (turquoise). The dashed vertical line indicates the skill items >50% of the respondents agreed were essential, which was considered some agreement.

## 4. Discussion

### 4.1. Limitations 

This study has several weaknesses. One is the selection of participants that contributed to the definition of EHL. Participants in this study were drawn from NIEHS and PEPH participants. From a participant perspective, the majority of participants were those with research positions, holding a Master’s degree or a PhD. Therefore, any findings would be biased towards what those in research would find essential to EHL. 

An additional bias is the geographic perspective; that is, the focus on US environmental health problems, largely of urban communities. Thus, the findings and the applicability of the definition are limited to the understanding of environmental impacts on human health to the United States and urban circumstances.

### 4.2. Environmental Health Literacy Knowledge and Skill Items

This study identified 32 knowledge and six skill items agreed upon by EH professionals as being essential for EHL and agreed upon by at least 50% of the respondents. In focusing on knowledge items, the team used a cluster of items which >70% of participants ranked as essential to identify the items that are strongly associated with EHL (Table 3). 

In examining these, we can see that there are knowledge items would not be covered under the concepts of SL or HL alone. Although item 6 is a key aspect of health literacy, where environmental agents can be analogous to smoking or salt intake, and item 4 is incorporated into science literacy, items 1–3 and 5 are unique to environmental health. Item 5 is of particular interest as it establishes the importance of agency and advocacy that is built into the history of environmental health exposures, and how mitigation of risk cannot be limited to an individual’s responsibility. Although this was not included in the 2014 definition of EHL, it is clear that EH professionals are in strong agreement that this knowledge is essential to EHL. 

The fact that there was not a similar level of strong agreement that skill items were essential to EHL is, in itself, an interesting finding. Although the definition of health literacy includes the capacity to process health information to make decisions, EHL, as defined by most EH professionals, does not appear to have made the leap from understanding to action. Even in examining those skill items with >50% agreement, the focus is on information seeking, versus information processing to action, with the exception of item 2, which speaks to one’s ability to communicate concerns to others (Table 4). 

In examining this cluster of skills, it does appear that science literacy is represented in item 6, which speaks to an individual’s ability to process fact from opinion. Health literacy is also represented in items 1 and 3–5, insofar as these speak to the capacity to obtain information to make health decisions. Again, we see a skill item associated with agency and advocacy in item 2, a skill that is not incorporated in HL or SL, which focus on individual decision making. 

### 4.3. Interconnectedness of Literacies/Environmental Health Literacy Concepts

The findings of this research indicate that EHL encompasses science and health literacy, but also includes some items unique to environmental health literacy. EHL goes beyond understanding proper care and treatment of an individual’s health conditions, to understanding the impact of exposures on one’s community, and the interplay between the environment and the individual. Some of these items fall under the currently accepted definition of EHL, whereas others do not, particularly those associated with advocacy.

Knowledge items related to science literacy, including exposure science and toxicology, were also frequently ranked as being essential. The meaning of science literacy has been debated since the 1950s, but is generally thought to mean a person can inquire, find, or determine answers to questions about curiosities experienced in everyday life, and evaluate the quality of scientific information on the basis of its sources and methods [26]. Items related to how contaminants enter the body, the frequency and duration of exposure, and modification of behaviors to mitigate exposures were agreed to be essential to EHL. Some key terminology was also identified as being essential to EHL. The terms risk, hazard, dose, and frequency of exposure were identified as key components of EHL. Some of these concepts move from more basic science into the field of risk assessment. Risk assessment is composed of hazard identification, exposure assessment, dose response, and risk characterization [37]. 

Items associated with science literacy, particularly the ability to separate fact from opinion, were also a recurring essential theme in EHL, including knowing that media coverage can be unbalanced; sensationalism is used in reporting environmental risks or hazards; and news headlines may not reflect the actual content of the article. 

The nature of the field of environmental health science carries with it perhaps greater uncertainty than other science focuses, because of the study of emerging contaminants. Knowledge of human health effects, prevalence in environment, and human susceptibility can be sparse, and media messages can be especially confusing and often contradictory [38]. This may explain the theme of science literacy dominating the agreed-upon skills associated with EHL. Environmental literacy has also included science literacy as being a significant component, especially because of a tendency of media to put a negative bias on emerging issues [39].

Knowledge of government involvement and policy regarding environmental exposures was also identified as being essential to EHL. Knowing that an individual can influence government policy is associated with political literacy and environmental justice [17,40,41]. Understanding that the presence of some environmental agents in commercial products, buildings, water, and air is regulated by government policy is more complex and unique to EHL. 

## 5. Conclusions and Implications for Research, Practice and Policy

Although the process of becoming environmentally health literate has already been defined as raising scientific literacy, environmental literacy, and numeracy, while increasing the awareness of specific exposures and their potential health effects, educational programs and community groups would benefit from a shared understanding of the skills and knowledge that underpin EHL. The aim of this study was to identify knowledge and skill items identified by EH professionals as being associated with EHL, and to understand how these items ranked by importance. 

The study resulted in two central findings. The first is that EH professionals had strong agreement (>70%) that a combination of knowledge associated with health and science literacy made up EHL, in addition to knowledge specific to environmental health, including advocacy. The identification of these six knowledge items associated with health literacy is helpful in establishing the basic knowledge that should precede any kind of engagement with communities around environmental exposures. The second is that EH professionals did not arrive at a strong agreement on skills that make up EHL, even though those skill items mirrored the incorporation of health and science literacy and advocacy. 

The findings of this study set the stage for future work on EHL. The identification of specific items of knowledge and skill that make up EHL can help educators and policy makers ensure that communities are set up to engage in discussions about exposures and how to mitigate risks. The issue of EHL should be addressed from two sides—education and accommodation—and this study has implications for both. 

From an educational perspective, establishing knowledge and skill becomes the basis for identifying clear learning objectives that can be shared with educators and outreach organizations. Given the limitation of this study in focusing on EH professionals associated with NIEHS and the PEPH network, the identification of these knowledge and skill items should only be considered a first step in establishing a basic curriculum for EHL. A similar study should include community members involved in grassroots advocacy and environmental justice initiatives. Although we acknowledge this limitation, the identification of essential knowledge items sets the stage for further research that includes identifying a consequence of low versus high EHL, and evaluation of methods and materials designed to improve EHL. It may be that different audiences, such as rural or international populations, people with limited literacy, or those from various ethnicities, have different information needs and skills. Another research topic under examination is developing comprehensive measures of EHL, which we have begun to address with our most recent work [42,43], which aims to develop a process-focused instrument to measure environmental health literacy.

Educating individuals is a proactive step to ensure that communities are able to be active participants in addressing environmental risks. Until education creates an equitable forum for engagement, policymakers and community groups have to accommodate EHL to ensure that communities at risk of exposure can reasonably mitigate risk. We cannot wait for high-risk communities to reach a level of EHL before taking action. 

The lack of strong agreement among EH professionals about the skills that are essential to EHL, and the fact that where there is agreement, those skills are focused on information gathering, is suggestive that participation in risk mitigation is a complex task that cannot be easily undertaken at an individual level. 

It would be of interest to understand why there is low agreement regarding the skills that are essential to EHL, and if this lack of agreement is shared by community stakeholders, and to establish the responsibility of industries and policy makers to ensuring that communities understand their exposure and their ability to engage in risk mitigation. There are many tools that public health promotion employs to achieve community understanding, including websites, print materials, public presentations, social media, videos, and hands-on/minds-on activities with youth and children.

This study suggests that EH professionals are in agreement that there is basic knowledge that the examined tools should convey, and that at minimum should: (1) include knowledge of the interplay between environment and health; (2) build on existing knowledge of how the environment impacts health, in addition to differentiating fact from opinion; and (3) inform individuals of how they are capable of influencing policy.

## Figures and Tables

**Table 3:** Knowledge Items chosen for the EHL Definition.

There Was >70% Agreement That a Good EHL Person Needs to Know (Knowledge):	
1	Knows that “environment” includes everything around us: air, water, man-made chemicals, natural chemicals, other people, culture, animals, food, microorganisms, and more.
2	Knows that people affect the health of the environment.
3	Knows that the environment affects every individual person’s health.
4	Knows that media coverage can be unbalanced.
5	Knows that an individual citizen can influence government policy.
6	Knows that the environment can affect a person’s health through exposure to many different environmental agents.

**Table 4:** Skill Items chosen for the EHL definition.

There Was Some >50% Agreement That a Person with Good EHL Can (Skill):	
1	Is able to find info explaining how to reduce risks in their life
2	Is able to convey their concerns about environmental risk to others
3	Is able to find info about hazards in their microenvironment, such as at home or in the workplace
4	Is able to find info about regional/community environmental hazards/issues
5	Is able to identify well-known/ established hazards in their environment
6	Is able to judge whether an information source is reliable

## Data Availability

The data presented in this study are available on request from the corresponding author.

## Supplementary Materials

The following are available online at https://www.mdpi.com/article/10.3390/ijerph182111626/s1, Supplementary A: Defining Environmental Health Literacy—Interview Script; Supplementary B: Environmental Health Literacy Data Analysis.
