# Supplementary material for: Defining Environmental Health Literacy
Source: Int J Environ Res Public Health. 2021 Nov 5;18(21):11626. doi: 10.3390/ijerph182111626 (PMC8583686; doi:10.3390/ijerph182111626)
Supplement: Supplementary file 1 [file ijerph-18-11626-s001.zip › Supplementary A- Defining Environmental Health Literacy ΓÇô Interview Script.pdf]

Supplementary A: Defining Environmental Health Literacy – Interview Script

Questions:

Please identify race, ethnicity, age, and educational level.

Describe your current job:
- What do you do?
- How long have you been doing it?
- Why do you to do this job?

Describe how your professional work involves discussing information about environmental health to members of the public (patients, students, community members)?

- What are you talking about with them?

What other things do you consider an "issue/concern" in environmental health?

In what situations does a person encounter and "issue" of environmental health? For example:
- General assessment of one's EH?
- Adverse health outcomes that may be linked to the environment?
- Reading or hearing about EH news from media or other lay people?
- Reading or hearing about EH from a physician, governmental entity, or organization?

So far in our research we have found much like general literacy, there is a scale ranging from poor literacy to higher literacy. We hope to uncover the knowledge, skills, and processes that those on higher end of the scale would possess or undertake when making a decision about an EH issues.

(Further analogy if necessary: In general literacy, there exists a large dictionary and a large book of grammar- however, it is not necessary to know all of these things to be considered literate. We would like to learn what information and skills boost a lay person from lower parts of the scale to higher scale- what do you want the higher scaled layperson to know and be able to do when they are faced with an EH issue?)
- What knowledge does the higher scaled individual possess?
    - How specific and detailed is this information? (give example: Do pregnant women just need to know they shouldn't eat lots of tuna, or should they know about mercury and what it does OR Do people need to know that microwaving in plastic is bad, or do they need to know about the chemicals and why heating makes them come out and what they do in the body)

    - How does this person approach a topic that they do not have prior knowledge about?
        - (Further analogy if necessary: We are hearing from other professionals that there is a collection of background knowledge that one needs to have filed to consider this information and make an educated decision- we have dubbed this 'the black box'. In your opinion what is in this box? Please be as specific as possible- if they understand the connection between their body and the environment…what should they know about their body? If they need basic science, what kind of science (biology)- to what level of detail (need to organelles)? What major concepts do they need to be aware of?
- What skills does the higher scaled individual possess?
    - What are the higher scaled individuals able to do with information?
    - What specific things do they do to analyze information?
    - Is there a process by which they come to a conclusion about the issue?
        - Are there specific questions or major things they consider about sources?
