# Supplementary material for: Defining Environmental Health Literacy
Source: Int J Environ Res Public Health. 2021 Nov 5;18(21):11626. doi: 10.3390/ijerph182111626 (PMC8583686; doi:10.3390/ijerph182111626)
Supplement: Supplementary file 1 [file ijerph-18-11626-s001.zip › Supplementary B- Environmental Health Literacy Data Analysis.pdf]

Supplementary B: Environmental Health Literacy Data Analysis

Isaac Jenkins, MS, Statistician
Southwest Environmental Health Sciences Center, Data Science Facility Core
March 27, 2014

1 Data Summary

Data are read from the file `EHLSurvey2014 DATA 2014-03-25 1627.csv` using the REDCap generated data import script `EHLSurvey2014 DATA 2014-03-25 1627.r`. The data has 281 rows and 338 columns. These represent 281 responses to the Environmental Health (EH) Literacy Survey. The survey asks whether 105 EH knowledge and skill items are essential to basic EH literacy or only required by experts, intermediates, or not at all. There are 66 completely blank responses. My REDCap experience leads me to believe these were caused by surveys being opened, but never started. These were removed from the analysis. There are 40 cases that have responses to fewer than 80% of the items. The client said these should be removed. This leaves 175 survey respondents.

2 Summary Plots

Below are net-stacked bar plots. They show, for each item, the percent of responses to each of four choices (Essential, Expert, Intermediate, and Not Included). The plots consider Essential as a positive response and the others as negative. In other words, Essential is required for basic EH literacy, and the other 3 choices give some indication that it is not. The numbers next to the bars indicate the item number. Unfortunately, there are no simple item identifiers in the survey, so the items were simply numbered from 1 to 105. Note that items 1 to 69 are knowledge-type items and items 70 to 105 are skill-type items. Plots are initially broken down by knowledge and skill items and later by the 13 categories of questions found in the survey (e.g., General Environmental Health Knowledge, Knowledge about exposures and environmental agents).

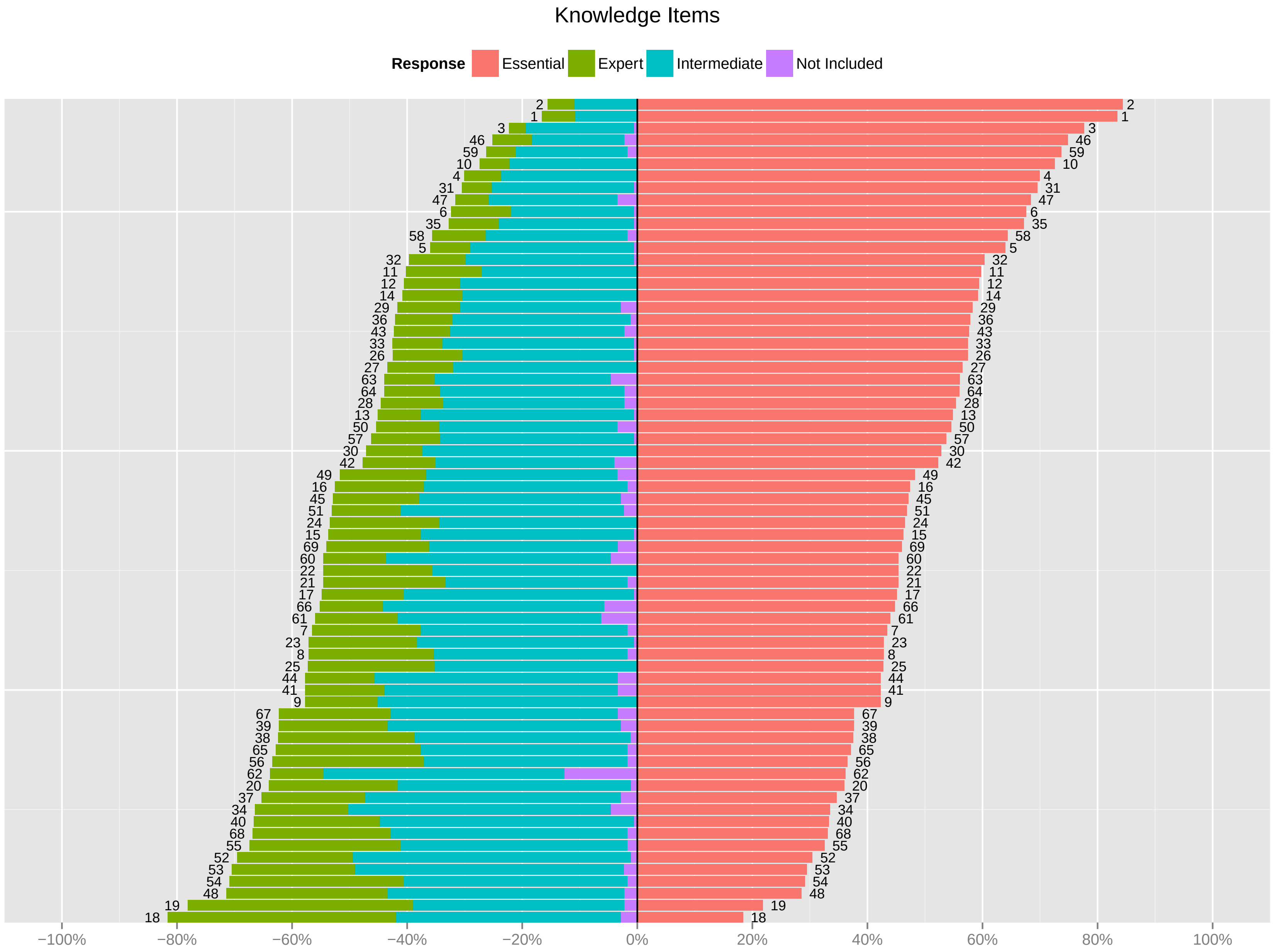

Figure 1: Percent of choice responses for knowledge-type items. Numbers next to the bar indicate the item number.

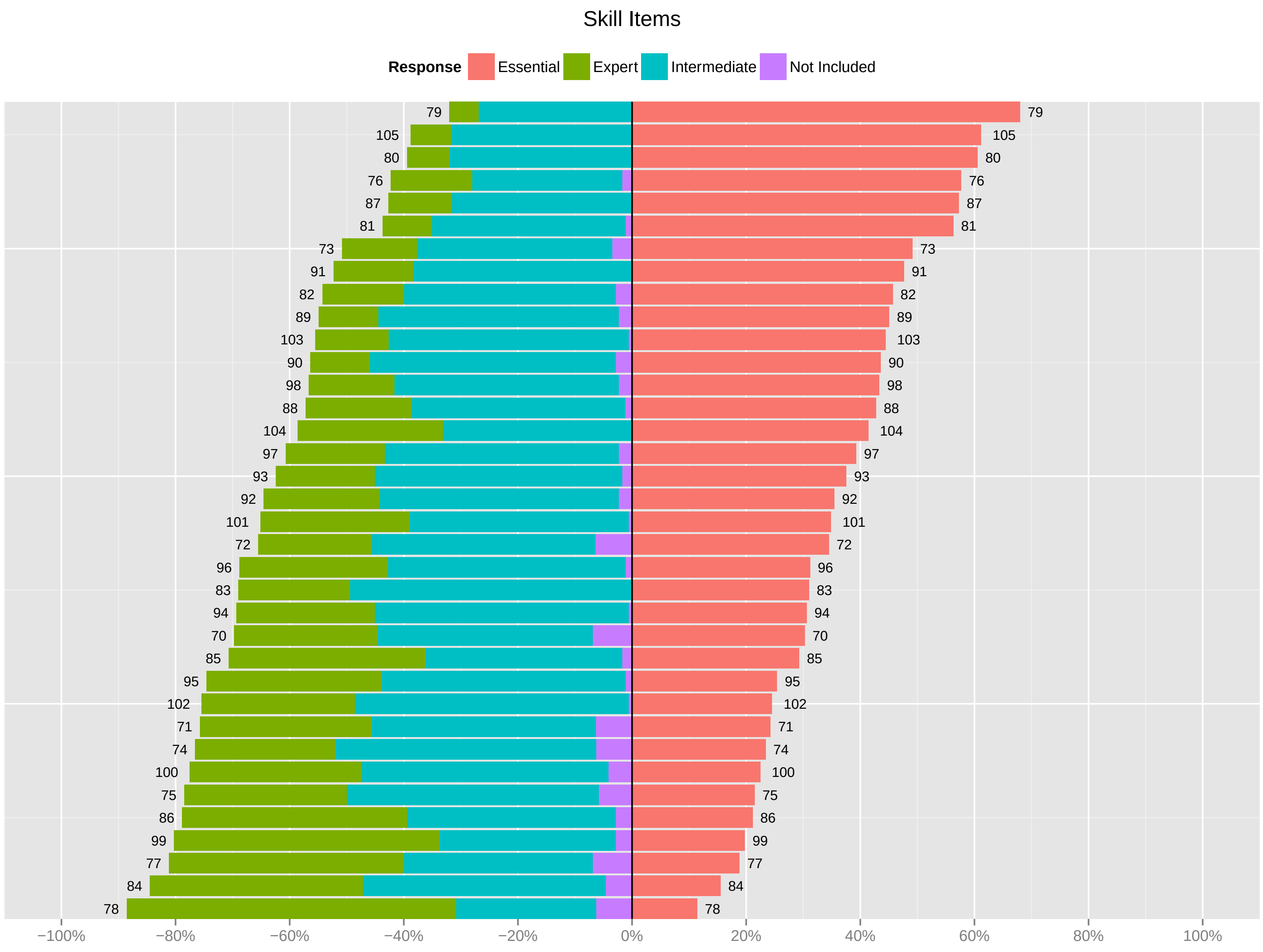

Figure 2: Percent of choice responses for skill-type items. Numbers next to the bar indicate the item number.

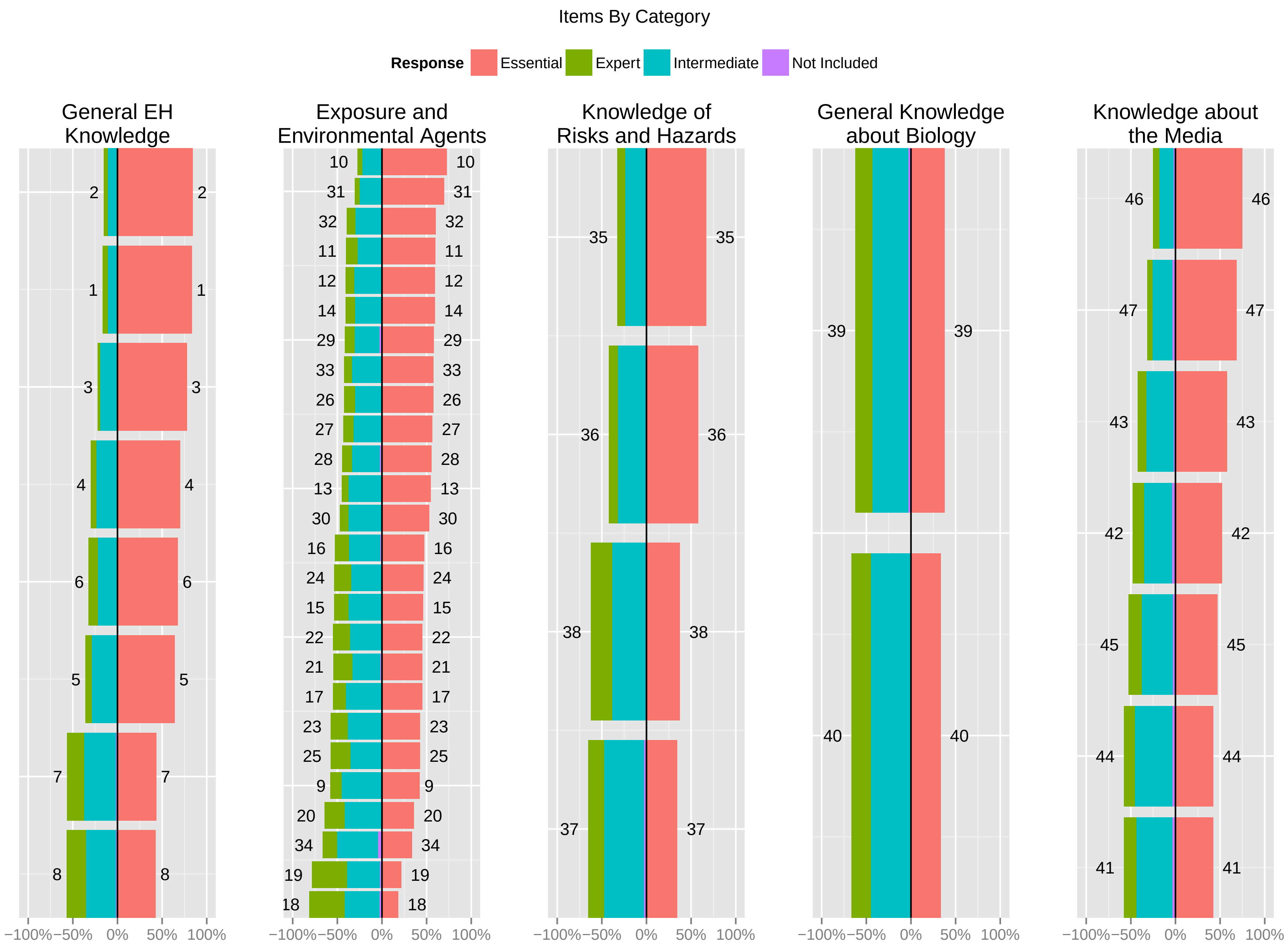

Figure 3: Percent of choice responses for each category of items. Numbers next to the bar indicate the item number.

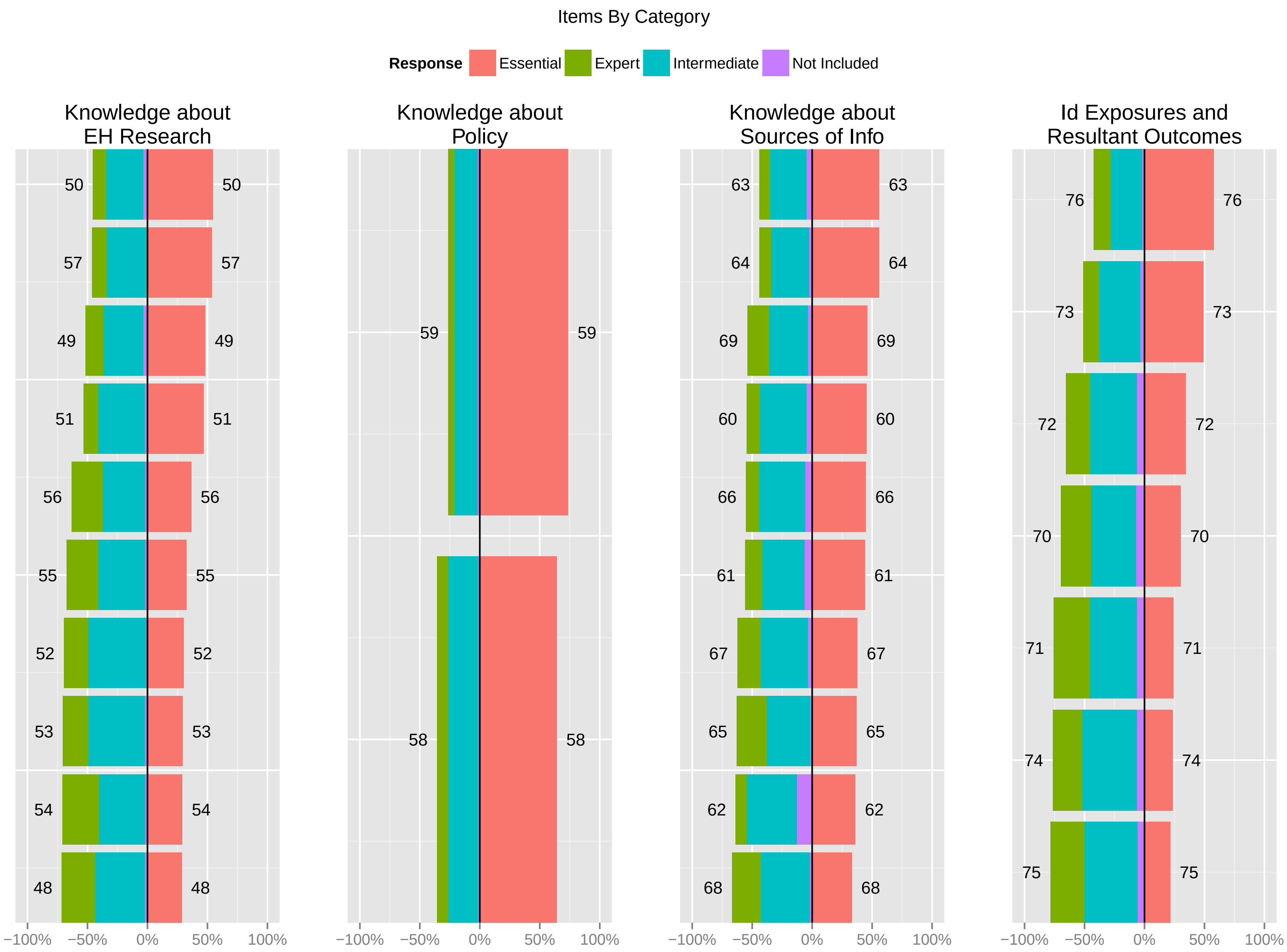

Figure 4: Percent of choice responses for each category of items. Numbers next to the bar indicate the item number.

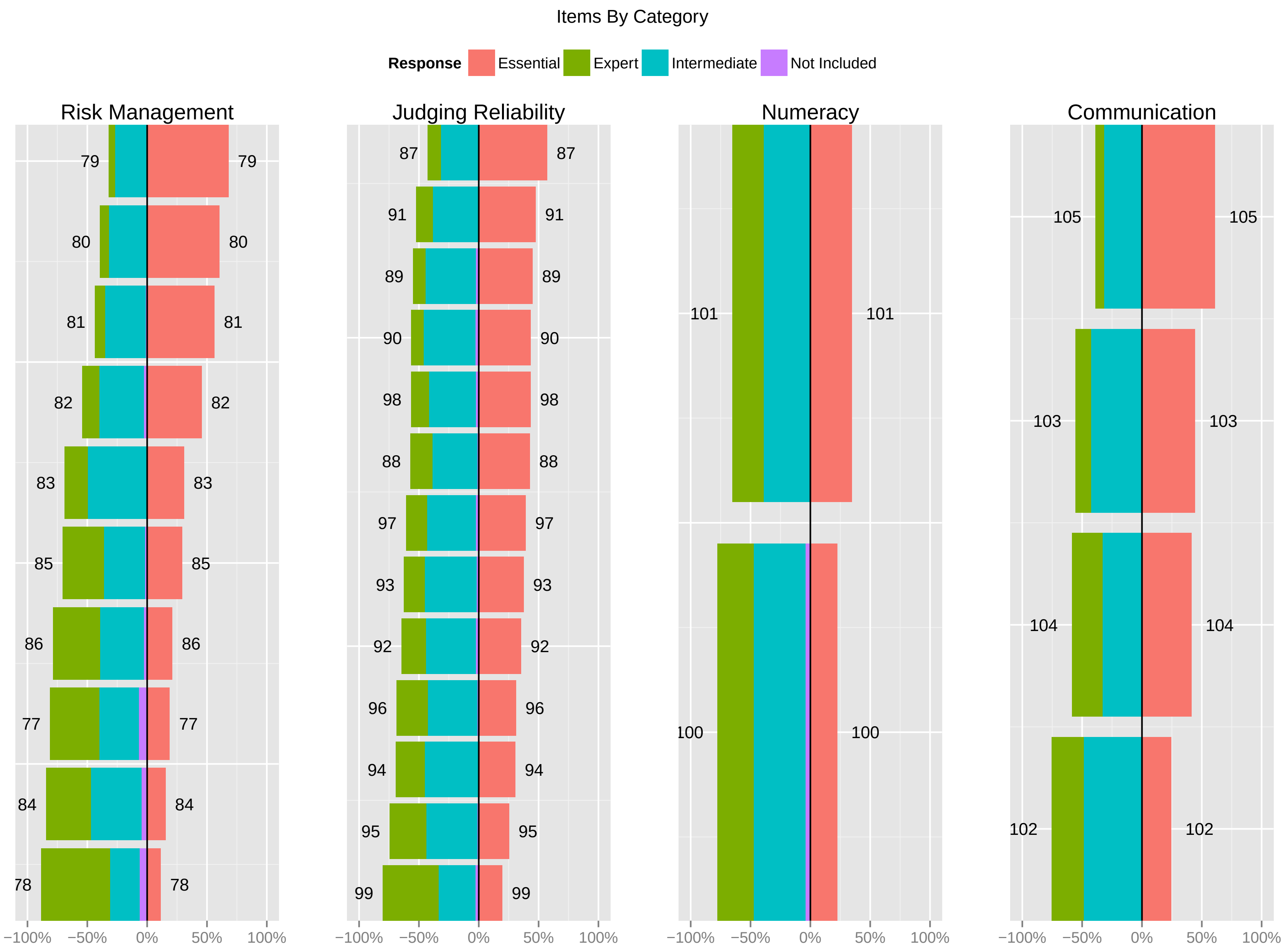

Figure 5: Percent of choice responses for each category of items. Numbers next to the bar indicate the item number.

3 Professions

Respondents were asked what their profession was. They were given 6 options. The distribution of responses are in table 1. Since there were only 3 medical doctors, these participants were shifted to the "Other Professions" category. Summary plots by knowedge- and skill-type items, as well as by item category are below.

Profession	n
Basic Science Researchers	29
Other Type of Researchers	42
Educators	36
EH Outreach Specialists	36
Medical Doctors	3
Other Professions	35

Table 1: Distibution of professions in the EH Literacy Survey

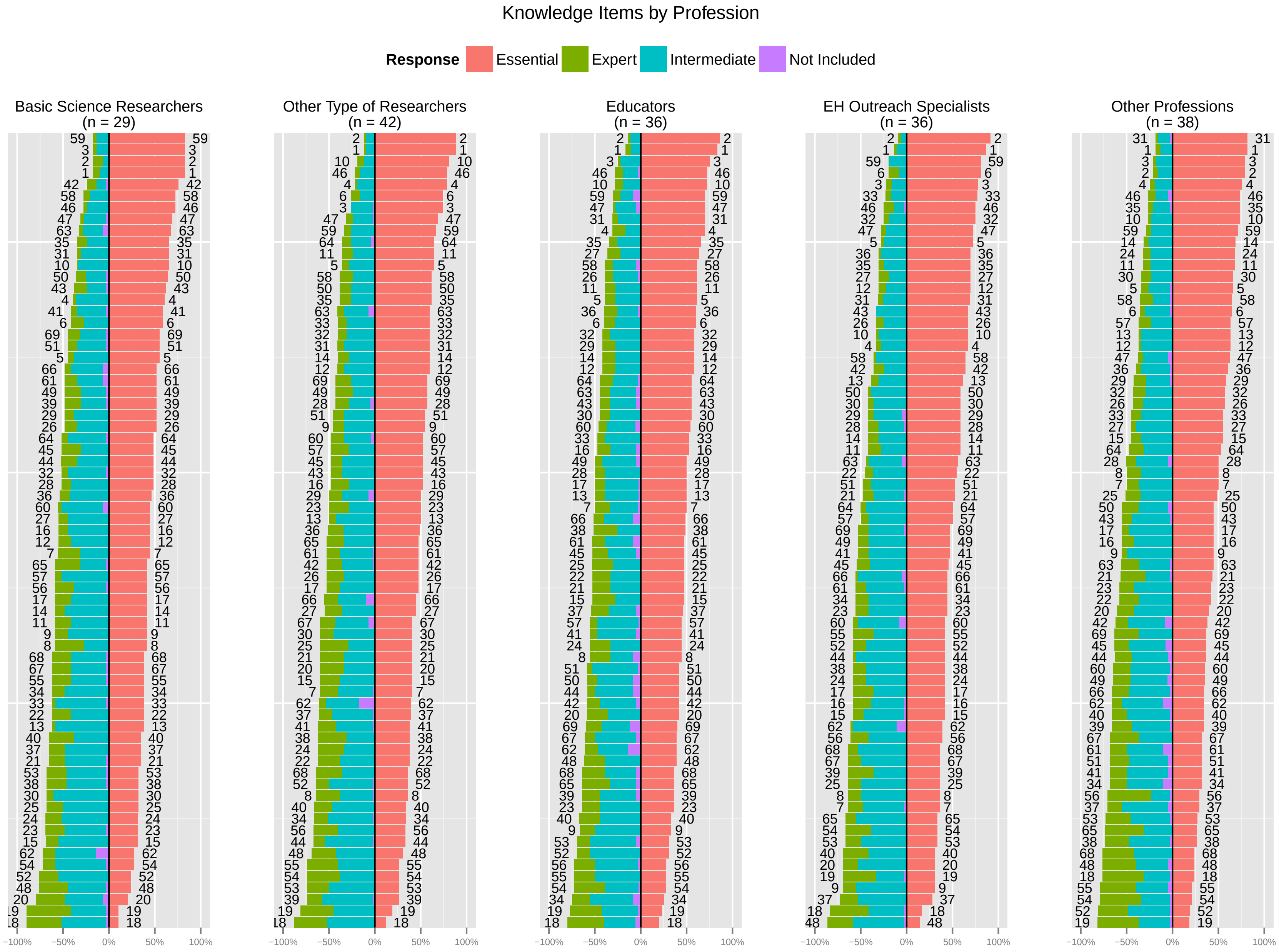

Figure 6: Percent of choice responses for knowledge-type items. Numbers next to the bar indicate the item number.

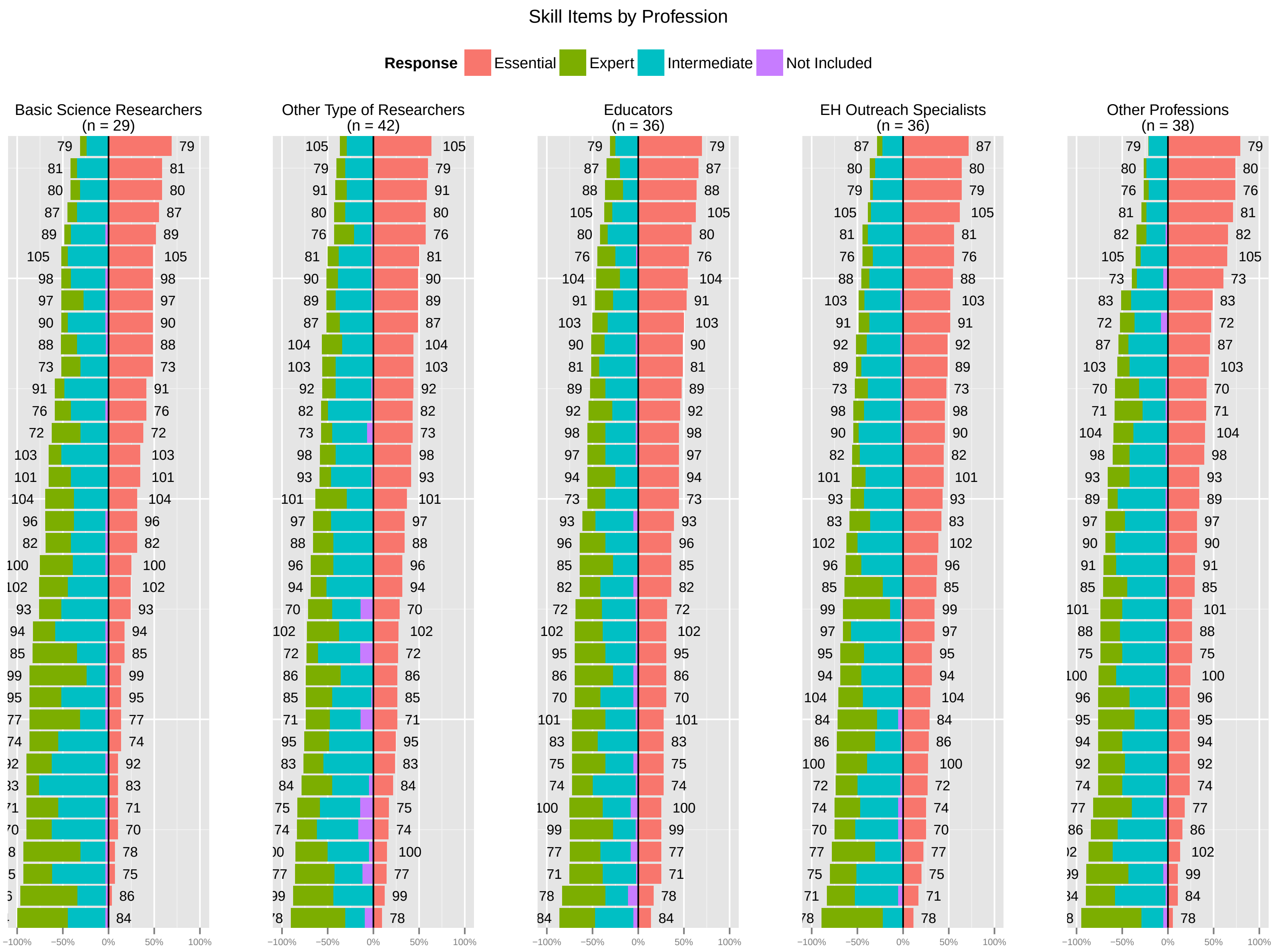

Figure 7: Percent of choice responses for skill-type items. Numbers next to the bar indicate the item number.

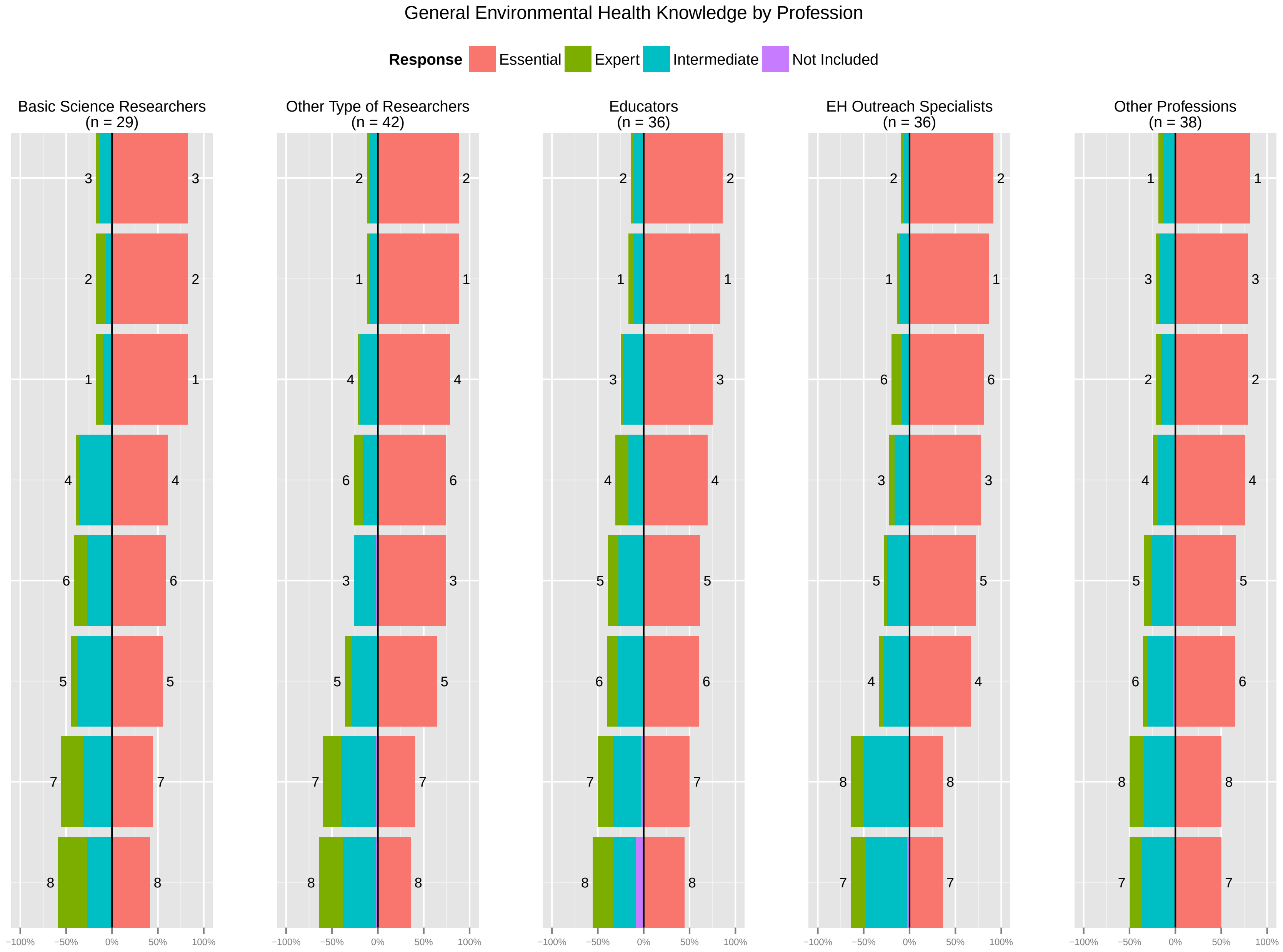

Figure 8: Percent of choice responses for each category of items. Numbers next to the bar indicate the item number.

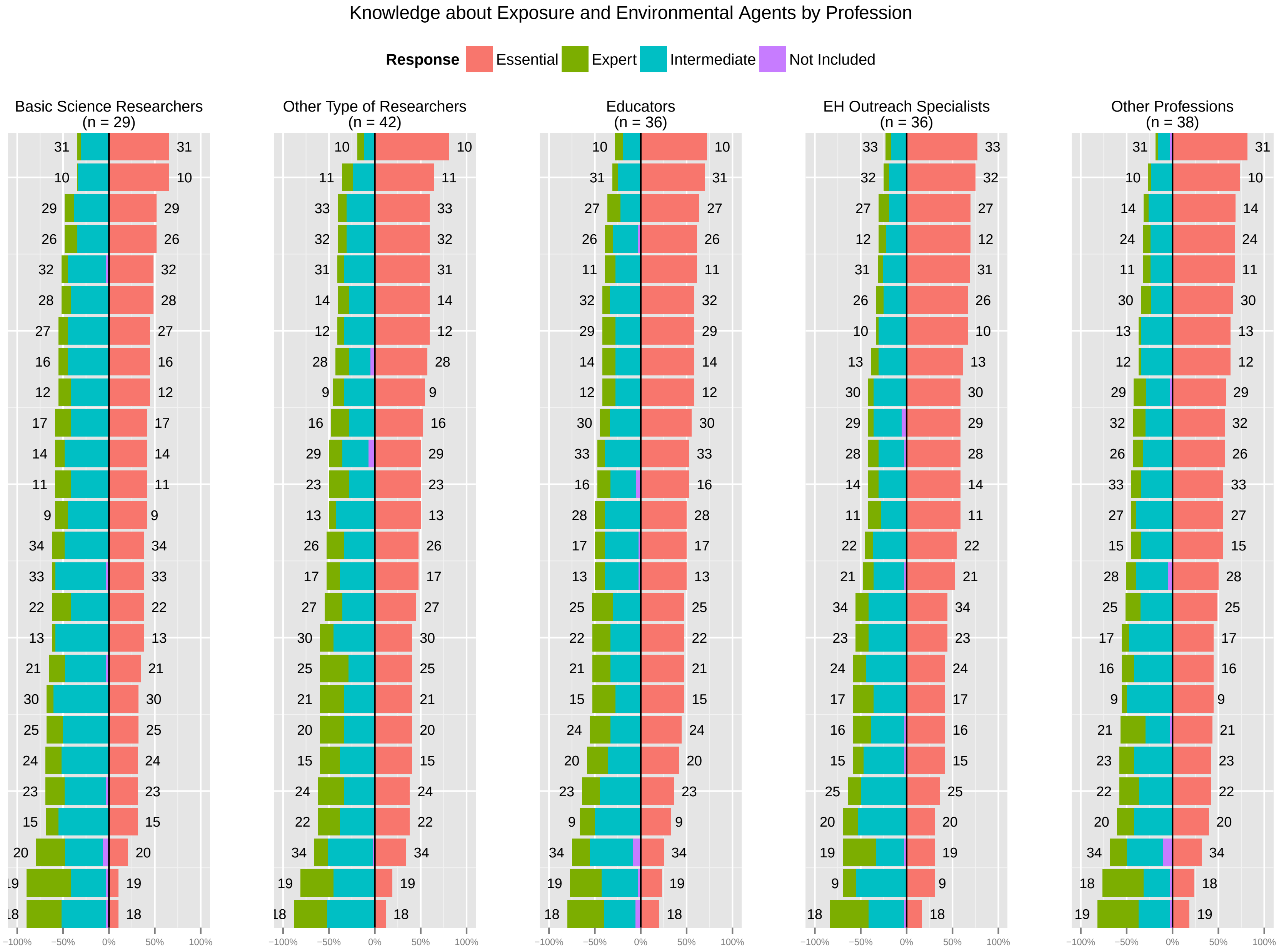

Figure 9: Percent of choice responses for each category of items. Numbers next to the bar indicate the item number.

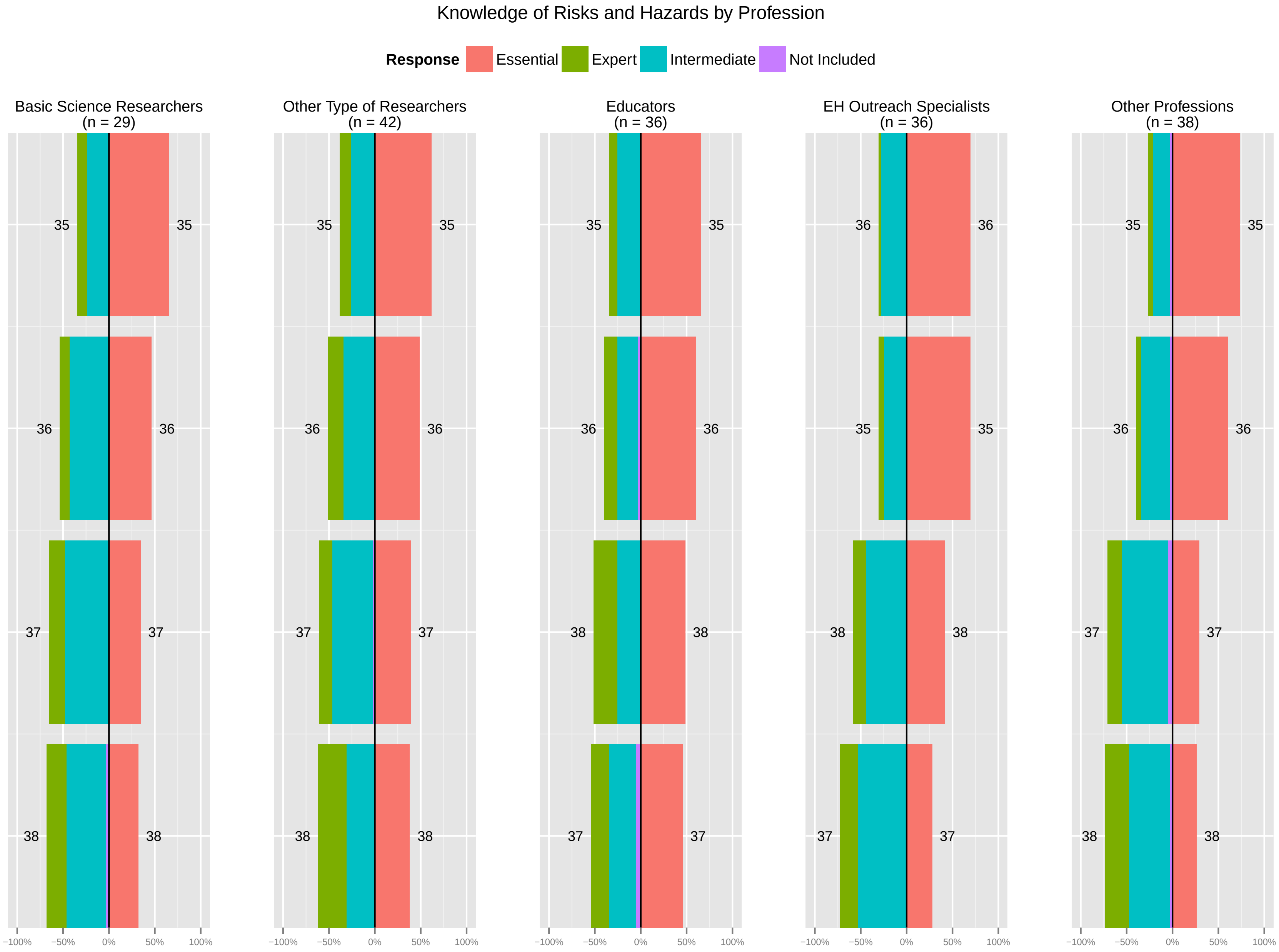

Figure 10: Percent of choice responses for each category of items. Numbers next to the bar indicate the item number.

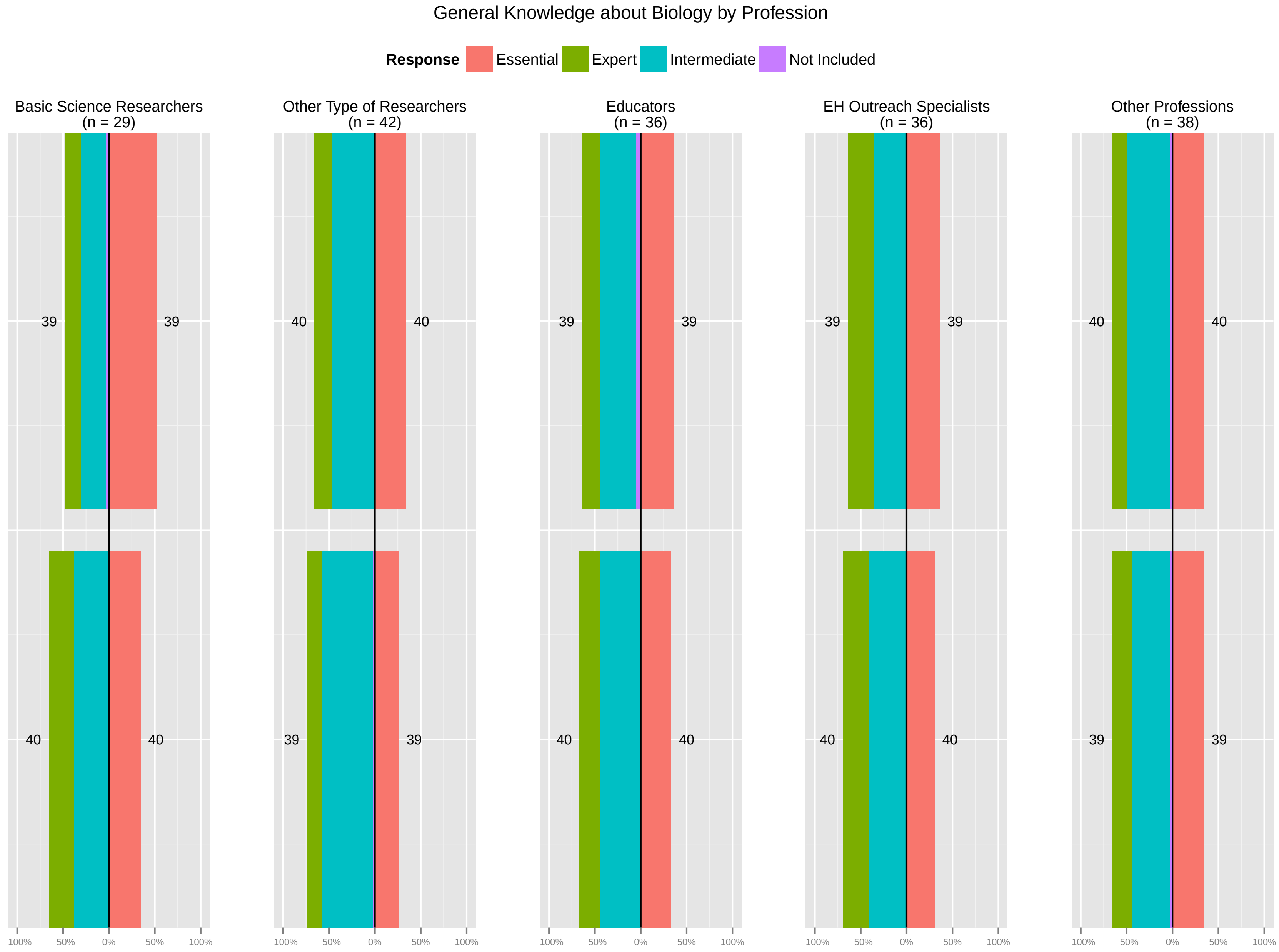

Figure 11: Percent of choice responses for each category of items. Numbers next to the bar indicate the item number.

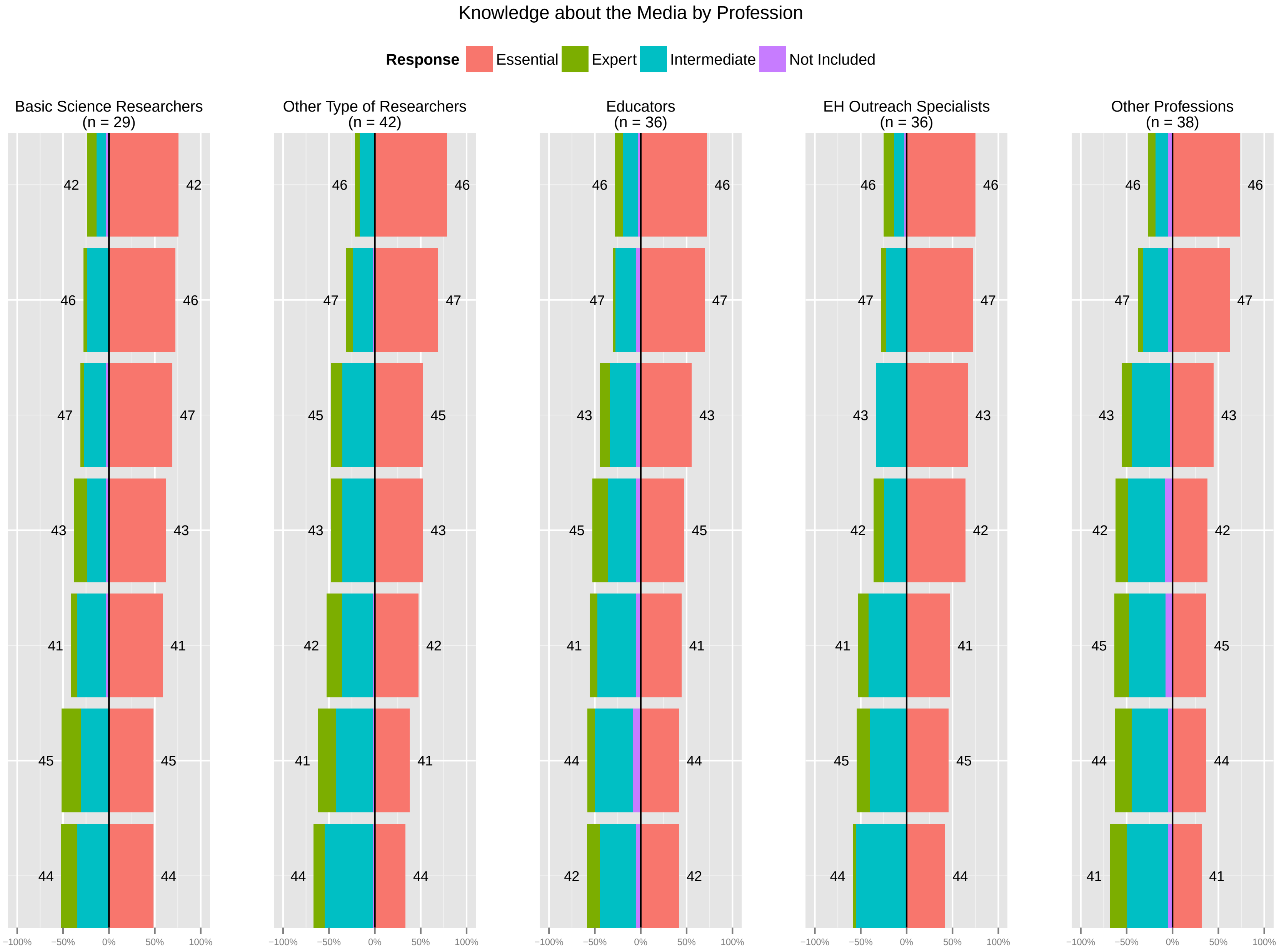

Figure 12: Percent of choice responses for each category of items. Numbers next to the bar indicate the item number.

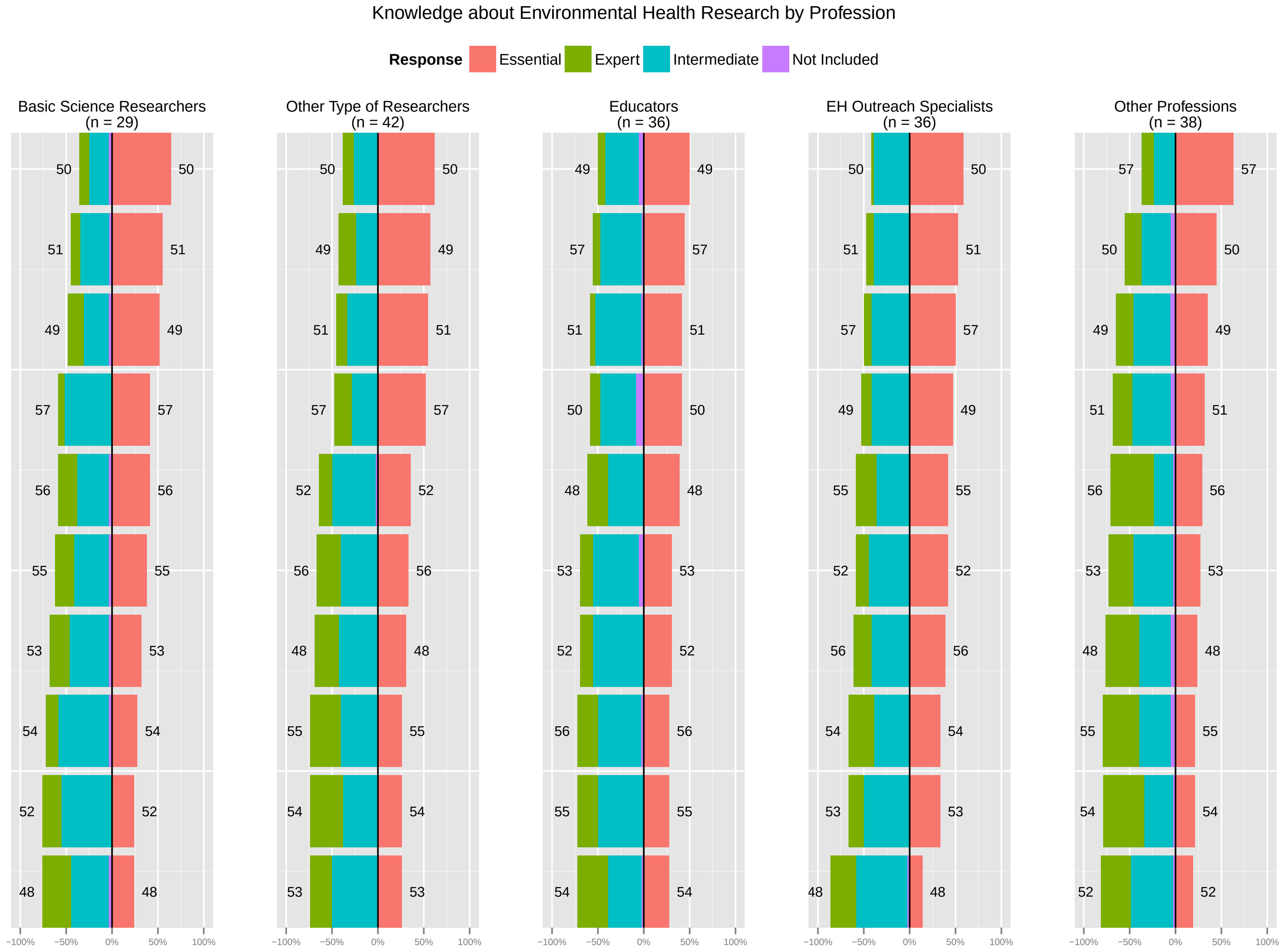

Figure 13: Percent of choice responses for each category of items. Numbers next to the bar indicate the item number.

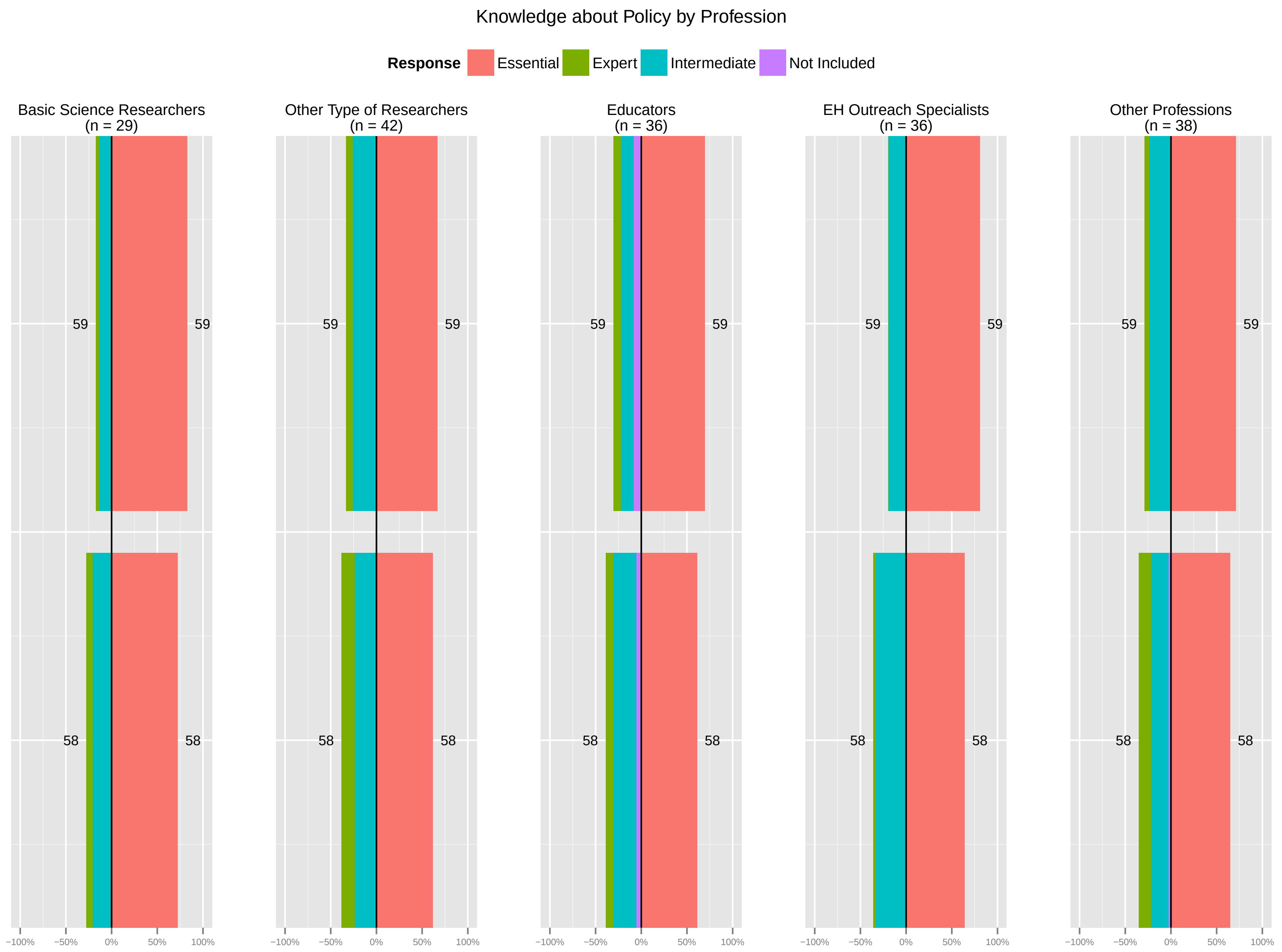

Figure 14: Percent of choice responses for each category of items. Numbers next to the bar indicate the item number.

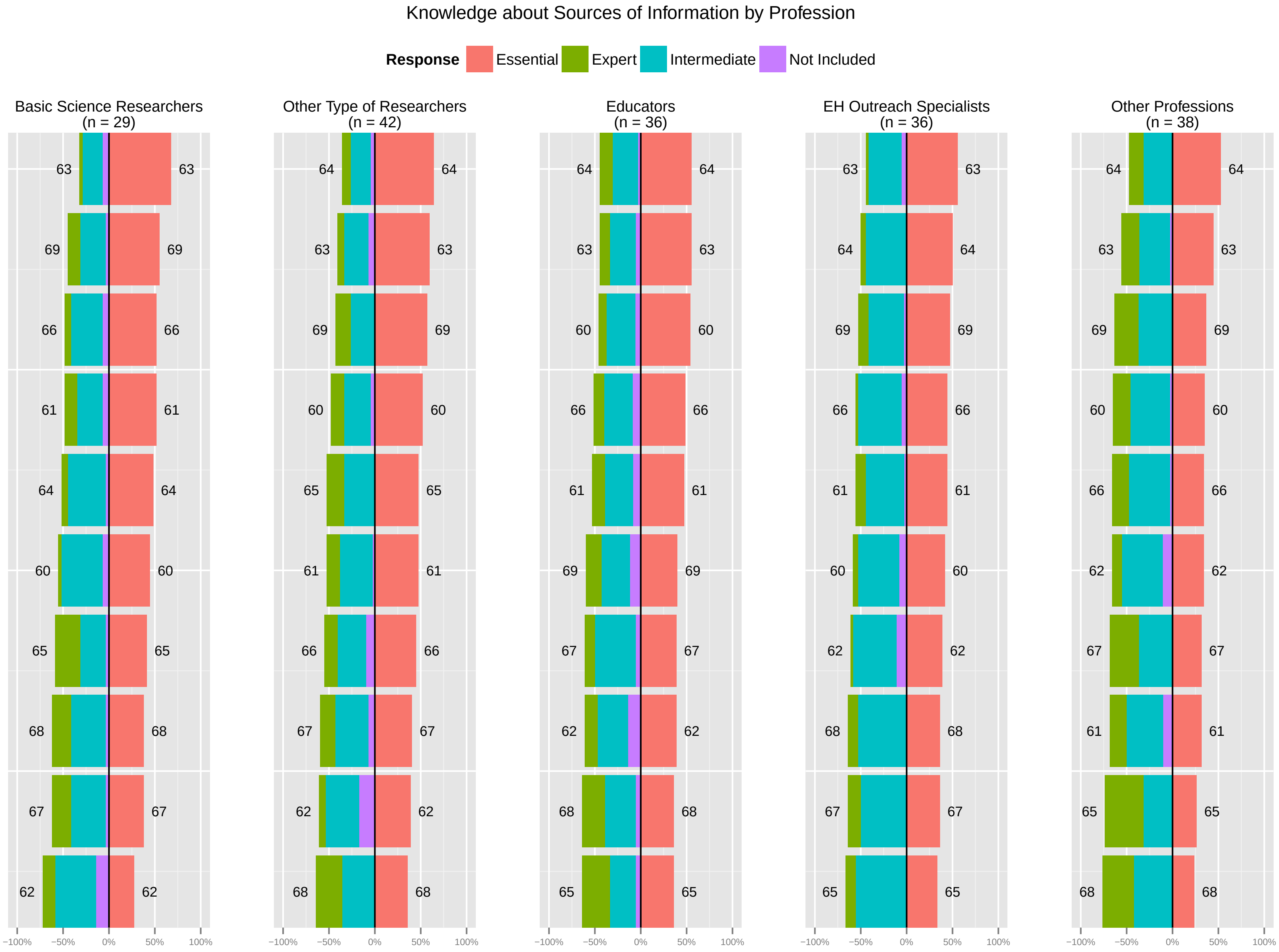

Figure 15: Percent of choice responses for each category of items. Numbers next to the bar indicate the item number.

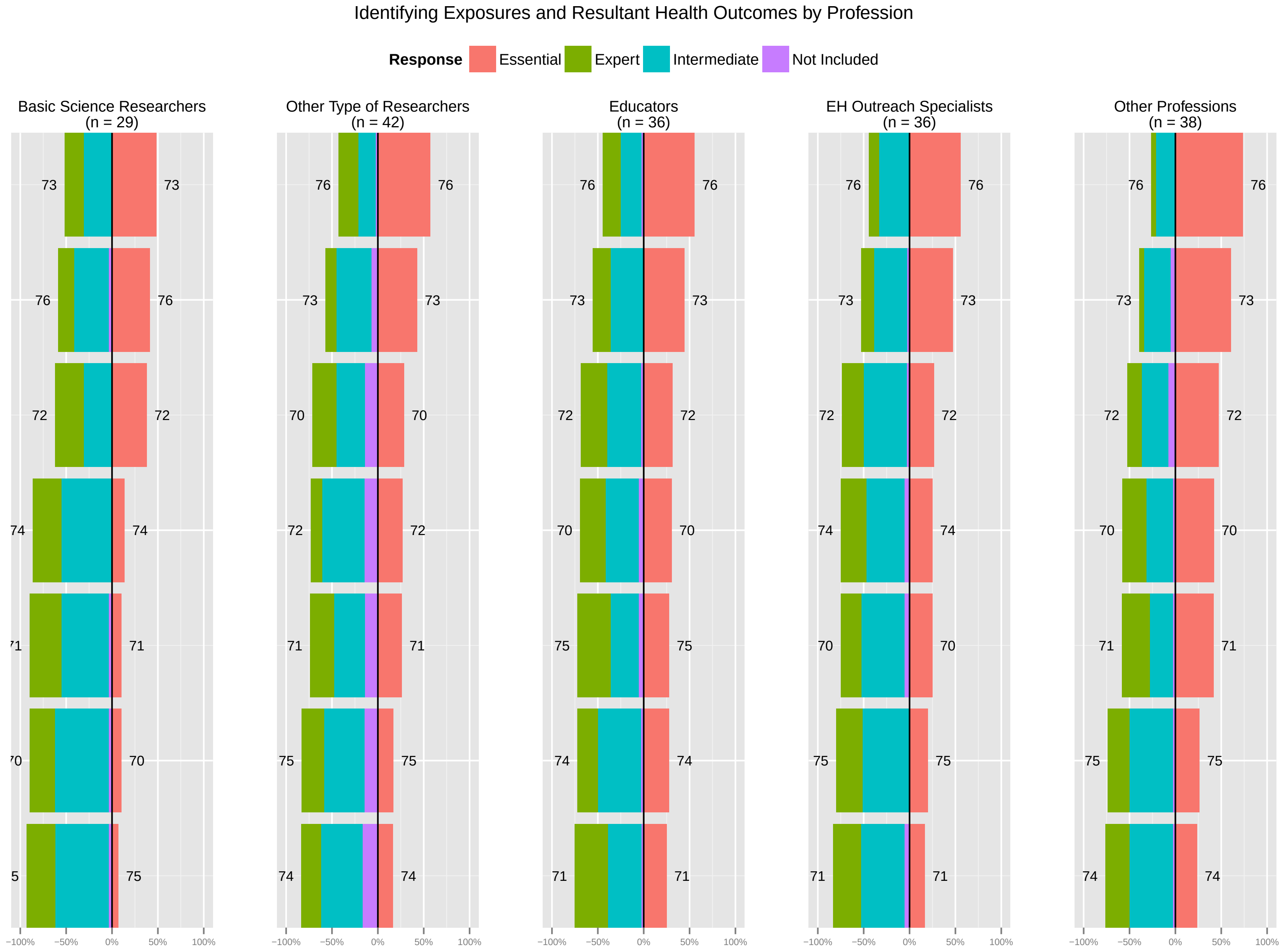

Figure 16: Percent of choice responses for each category of items. Numbers next to the bar indicate the item number.

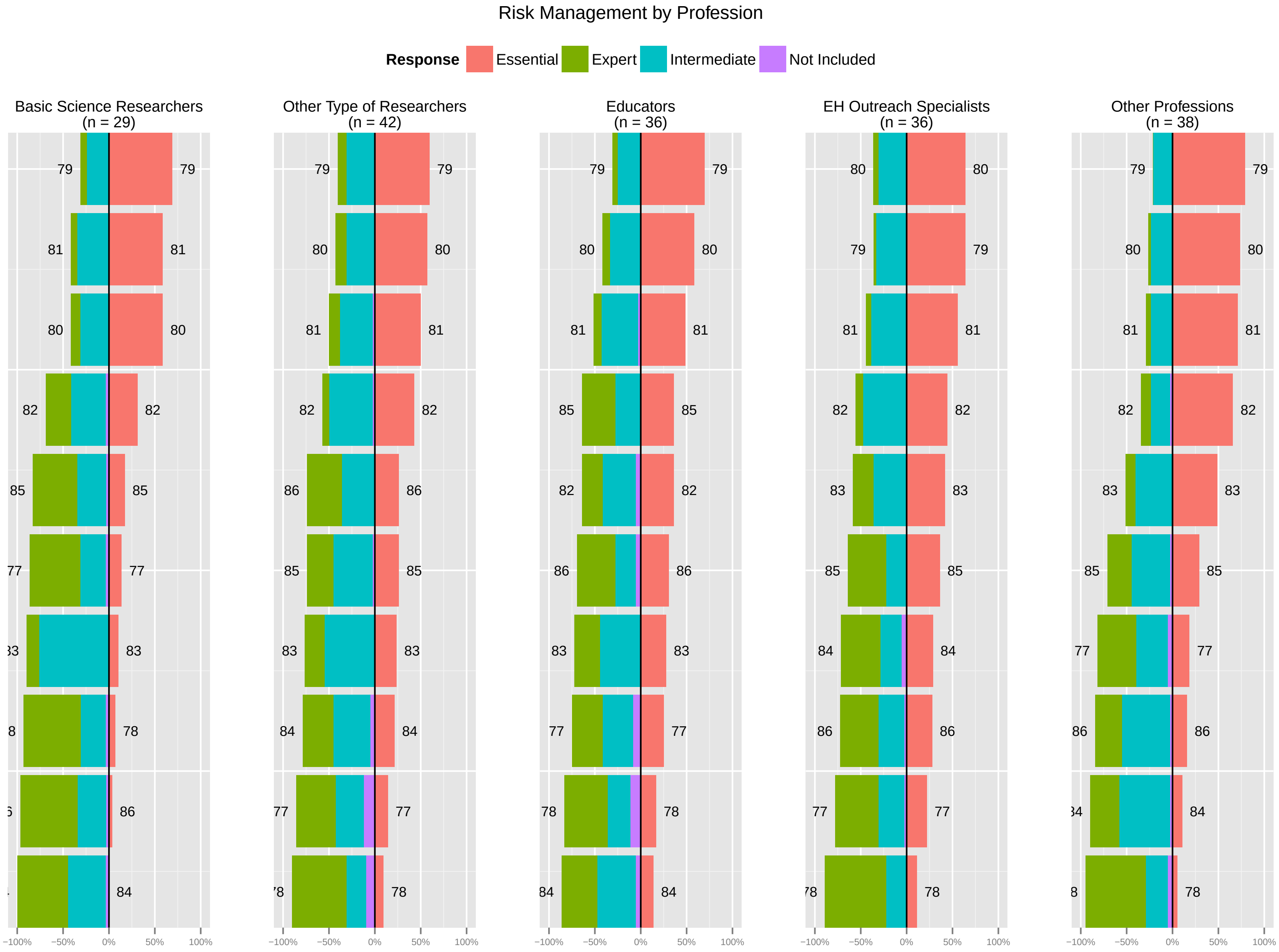

Figure 17: Percent of choice responses for each category of items. Numbers next to the bar indicate the item number.

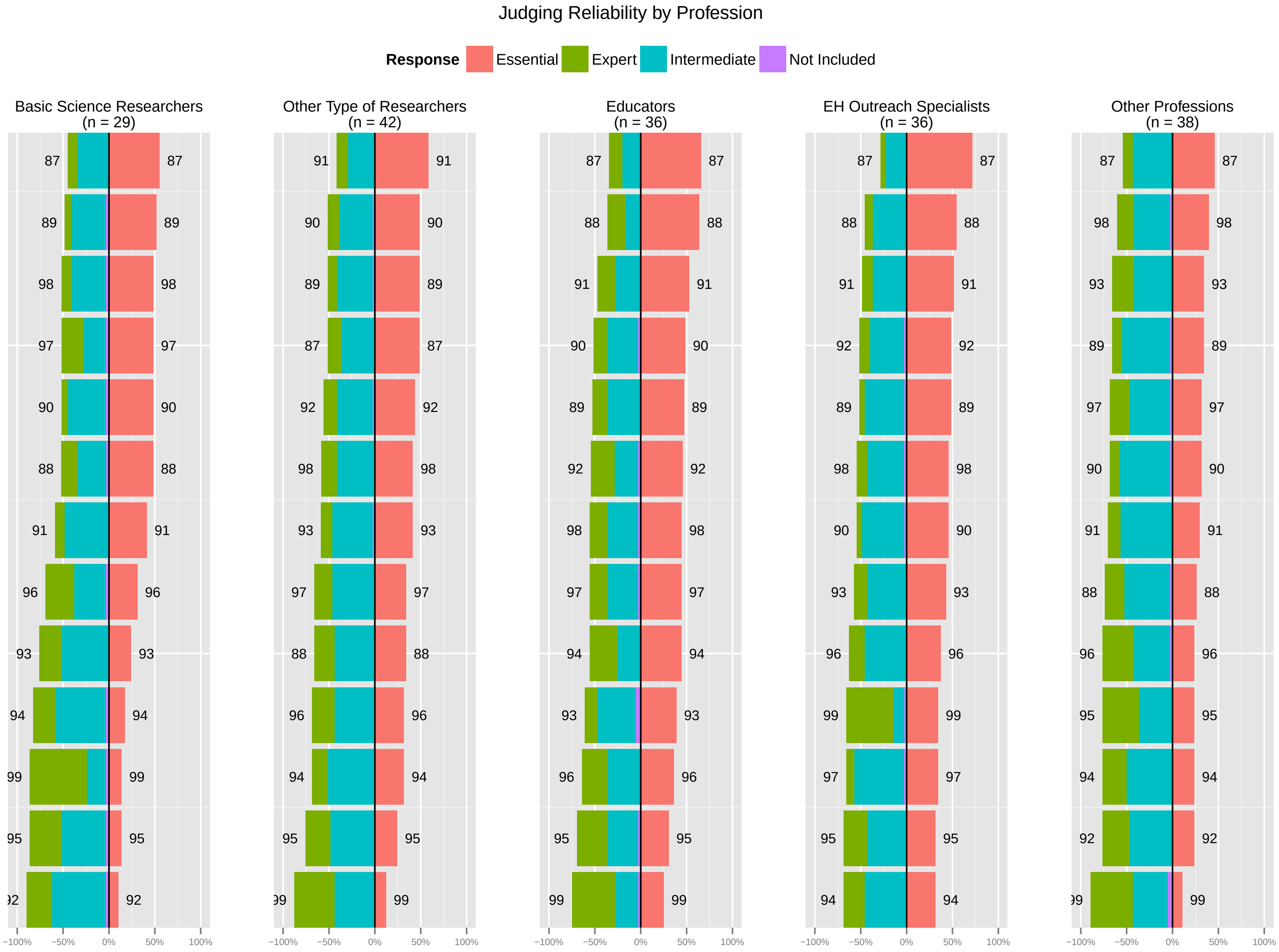

Figure 18: Percent of choice responses for each category of items. Numbers next to the bar indicate the item number.

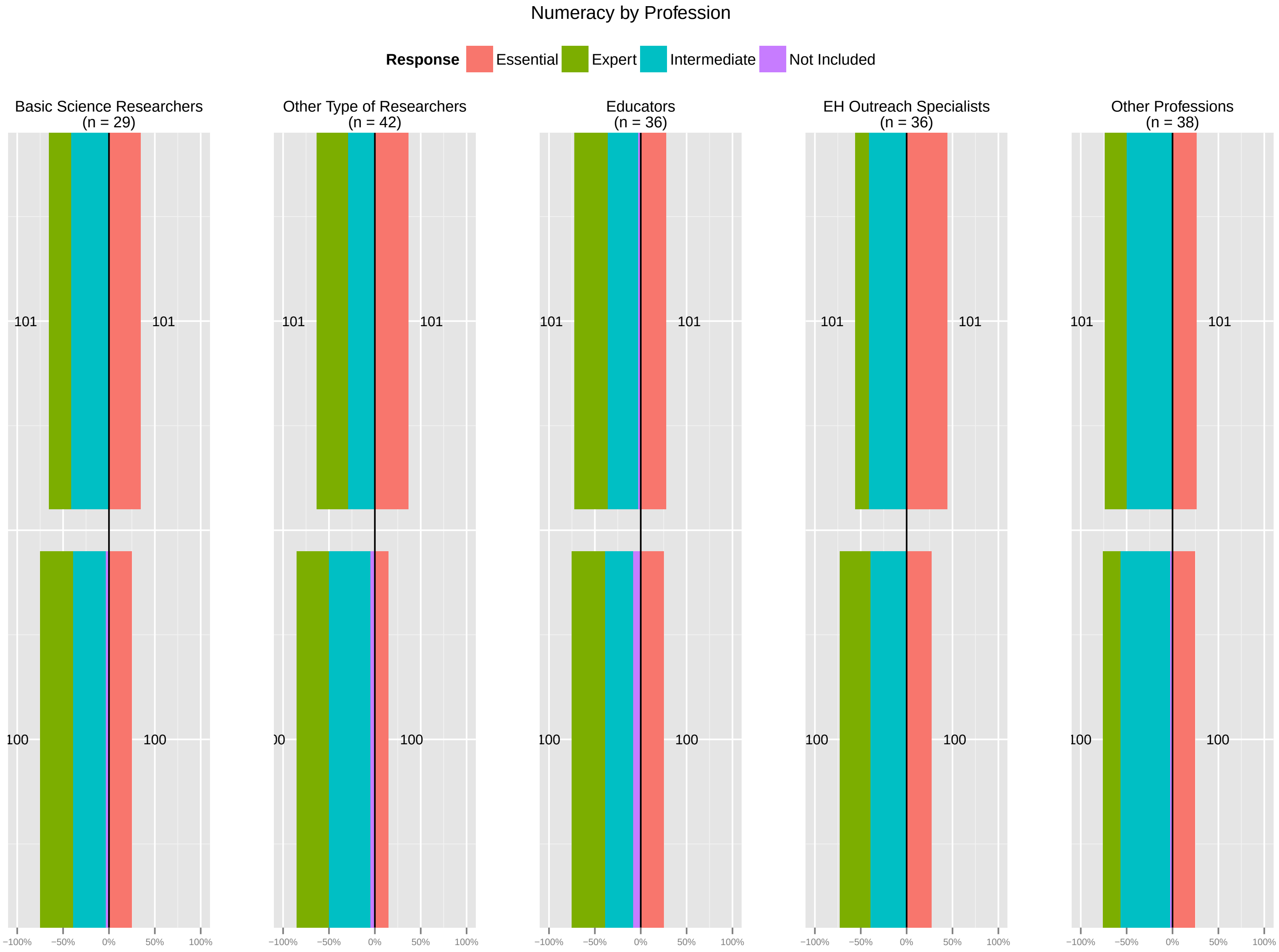

Figure 19: Percent of choice responses for each category of items. Numbers next to the bar indicate the item number.

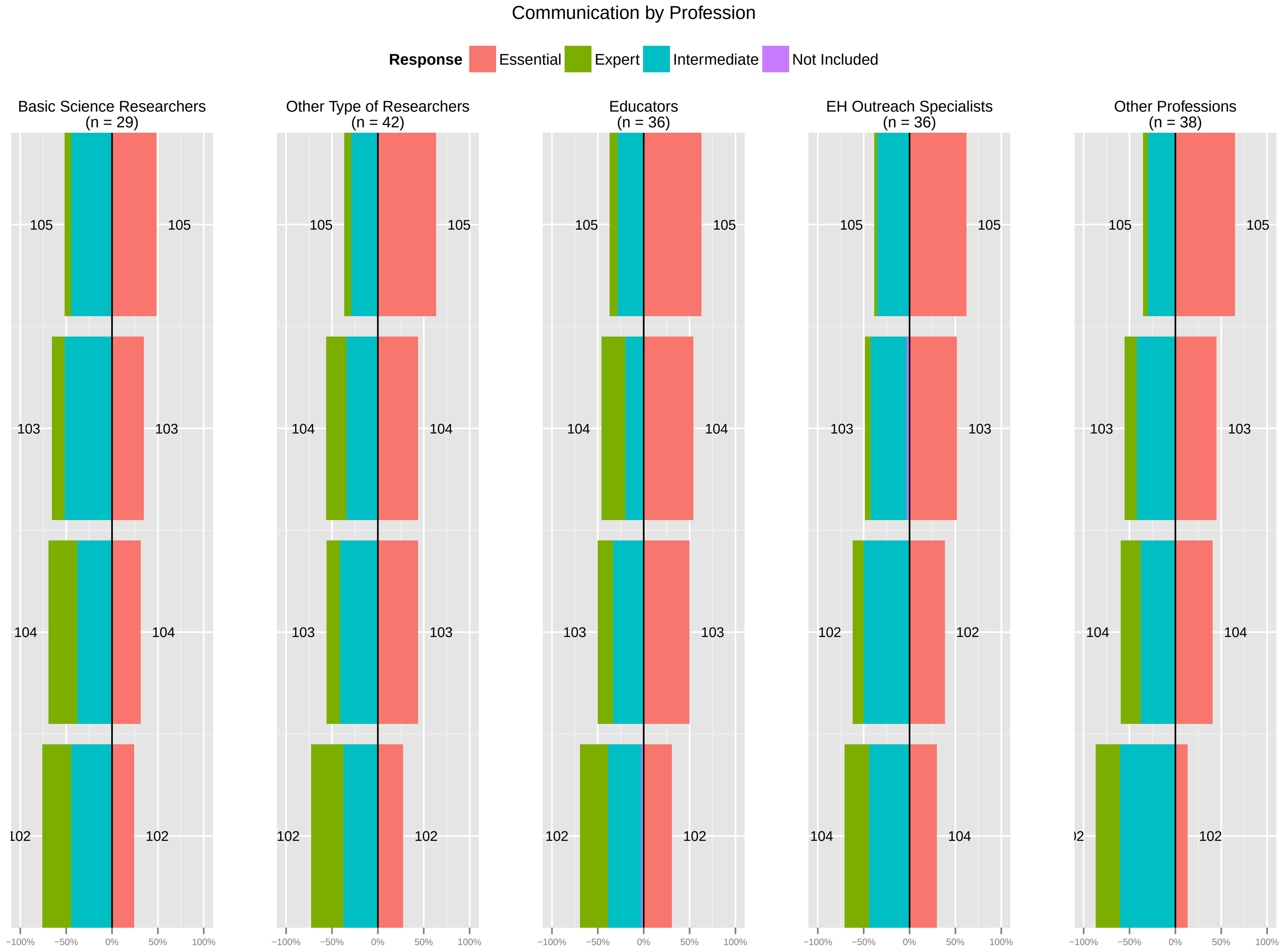

Figure 20: Percent of choice responses for each category of items. Numbers next to the bar indicate the item number.

Appendices

A R Session Information

```
R version 3.0.2 (2013-09-25)
Platform: x86_64-apple-darwin10.8.0 (64-bit)

locale:
[1] en_US.UTF-8/en_US.UTF-8/en_US.UTF-8/C/en_US.UTF-8/en_US.UTF-8

attached base packages:
[1] splines   grid      stats     graphics  grDevices utils     datasets
[8] methods   base

other attached packages:
 [1] xtable_1.7-1    gridExtra_0.9.1 dplyr_0.1.3     scales_0.2.3
 [5] ggplot2_0.9.3.1 reshape2_1.2.2  Hmisc_3.14-3    Formula_1.1-1
 [9] survival_2.37-7 lattice_0.20-27 knitr_1.5

loaded via a namespace (and not attached):
 [1] assertthat_0.1      cluster_1.14.4      codetools_0.2-8
 [4] colorspace_1.2-4    dichromat_2.0-0     digest_0.6.4
 [7] evaluate_0.5.1      formatR_0.10        gtable_0.1.2
[10] highr_0.3           labeling_0.2        latticeExtra_0.6-26
[13] MASS_7.3-29         munsell_0.4.2       plyr_1.8.1
[16] proto_0.3-10        RColorBrewer_1.0-5  Rcpp_0.11.0
[19] stringr_0.6.2       tools_3.0.2
```
